# Classification and Multifaceted Potential of Secondary Metabolites Produced by *Bacillus subtilis* Group: A Comprehensive Review

**DOI:** 10.3390/molecules28030927

**Published:** 2023-01-17

**Authors:** Sajid Iqbal, Farida Begum, Ali A. Rabaan, Mohammed Aljeldah, Basim R. Al Shammari, Abdulsalam Alawfi, Amer Alshengeti, Tarek Sulaiman, Alam Khan

**Affiliations:** 1 Atta-ur-Rahman School of Applied Biosciences, National University of Sciences and Technology (NUST), Islamabad 44000, Pakistan; 2 Department of Biochemistry, Abdul Wali Khan University Mardan (AWKUM), Mardan 23200, Pakistan; 3 Molecular Diagnostic Laboratory, Johns Hopkins Aramco Healthcare, Dhahran 31311, Saudi Arabia; 4 College of Medicine, Alfaisal University, Riyadh 11533, Saudi Arabia; 5 Department of Public Health and Nutrition, The University of Haripur, Haripur 22610, Pakistan; 6 Department of Clinical Laboratory Sciences, College of Applied Medical Sciences, University of Hafr Al Batin, Hafr Al Batin 39831, Saudi Arabia; 7 Department of Pediatrics, College of Medicine, Taibah University, Al-Madinah 41491, Saudi Arabia; 8 Department of Infection Prevention and Control, Prince Mohammad Bin Abdulaziz Hospital, National Guard Health Affairs, Al-Madinah 41491, Saudi Arabia; 9 Infectious Diseases Section, Medical Specialties Department, King Fahad Medical City, Riyadh 12231, Saudi Arabia; 10 Department of Life Sciences, Abasyn University Islamabad Campus, Islamabad 44000, Pakistan

**Keywords:** *Bacillus subtilis*, secondary metabolites, non-ribosomal peptides (NRPs), ribosomal peptides (RPs) polyketides (PKs), hybrid NRPs/PKs, applications

## Abstract

Despite their remarkable biosynthetic potential, *Bacillus subtilis* have been widely overlooked. However, their capability to withstand harsh conditions (extreme temperature, Ultraviolet (UV) and γ-radiation, and dehydration) and the promiscuous metabolites they synthesize have created increased commercial interest in them as a therapeutic agent, a food preservative, and a plant-pathogen control agent. Nevertheless, the commercial-scale availability of these metabolites is constrained due to challenges in their accessibility via synthesis and low fermentation yields. In the context of this rising in interest, we comprehensively visualized the antimicrobial peptides produced by *B. subtilis* and highlighted their prospective applications in various industries. Moreover, we proposed and classified these metabolites produced by the *B. subtilis* group based on their biosynthetic pathways and chemical structures. The biosynthetic pathway, bioactivity, and chemical structure are discussed in detail for each class. We believe that this review will spark a renewed interest in the often disregarded *B. subtilis* and its remarkable biosynthetic capabilities.

## 1. Introduction

The *Bacillus subtilis* (*B. subtilis*) group is a ubiquitous Gram-positive bacteria with a remarkable adaptability potential that enables it to survive in highly diverse environments [1]. It is non-pathogenic and presents incredible genetic diversity even within closely related strains [2]. The *B. subtilis* group consists of four species, including *Bacillus subtilis*, *Bacillus pumilus*, *Bacillus licheniformis*, and *Bacillus amyloliquefaciens*, discovered several decades ago. Over time, numerous new species and subspecies have been described based on molecular evolution, physiology, and chemotaxonomic characterization [3]. *B. subtilis* is a model organism used to investigate cell motility, biofilm formation, protein secretion, cell division, secondary metabolites biosynthesis, adherence to plant root and fungal hyphae, cytoplasm altercation via intracellular nanotubes, and kin-recognition [4]. In biotechnological industries, *B. subtilis* is a popular workhorse used for the biosynthesis of a broad range natural products, from enzymes to purified bioactive compounds [5,6]. Its natural competence to genetic engineering and well-described gene expression system makes it attractive on many occasions [6]. Moreover, recently, it also earned attention as a biocontrol agent in agronomy by antagonizing phytopathogens and promoting plant growth [7].

**Figure 1:**
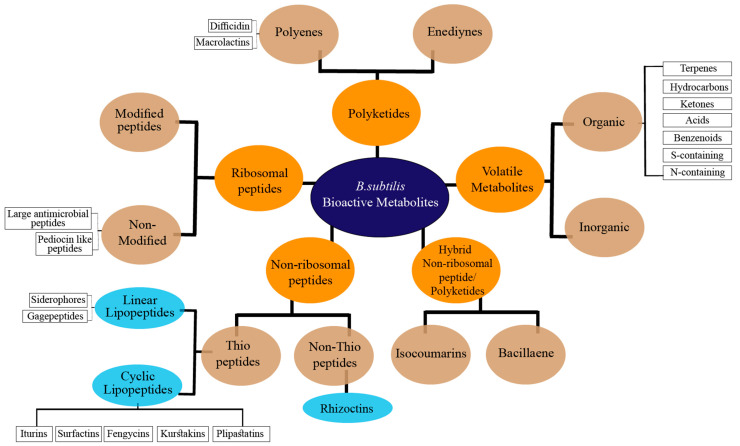
Classification of bioactive metabolites produced by *B. subtilis* complex.

The impressive skill set of *B. subtilis* for producing diverse bioactive metabolites was recognized in the last decade. It has been demonstrated that ~5% of a wild-type *B. subtilis* genome is exclusively devoted to the synthesis of bioactive compounds [8]. For a long time, it was considered for the production only of cyclic peptides such as iturins, surfactins, and fengycins [9,10]. Nonetheless, due to the discovery of numerous antimicrobial exhibiting linear lipopeptides, PKs, and volatile metabolites, it has gained a high commercial interest. The versatile bioactive metabolites produced by the *B. subtilis* group may be classified based on several criteria, including their biosynthetic pathways, function, structure, source, physicochemical properties, molecular targets, or bonding patterns [11]. Herein, we classified the bioactive metabolites from *B. subtilis* based on their molecular structures and biosynthetic pathways (Figure 1). In the current review, the bioactive metabolites produced by *B. subtilis* are classified into five classes, including non-ribosomal peptides (NRPs), polyketides (PKs), ribosomal peptides (RPs), and hybrid and volatile metabolites. These five classes are further categorized into various subclasses and described in detail.

Additionally, we comprehensively summarized all the reported bioactive metabolites produced by the *B. subtilis* group (Supplementary Material, Table S1–S5). Previous reports only focused on a single subclass or class, such as lipopeptides, lantibiotics, macrolides, and volatile metabolites [12,13,14,15]. Currently, in a time of drug resistance and high demand for eco-friendly pest control strategies, *B. subtilis* and its bioactive compounds could be the right choice. Therefore, we expect that this review will increase commercial interest in utilizing *B. subtilis* origin antimicrobial metabolites and biosurfactants in various industries.

## 2. Non-Ribosomal Peptides (NRPs)

**Figure 2:**
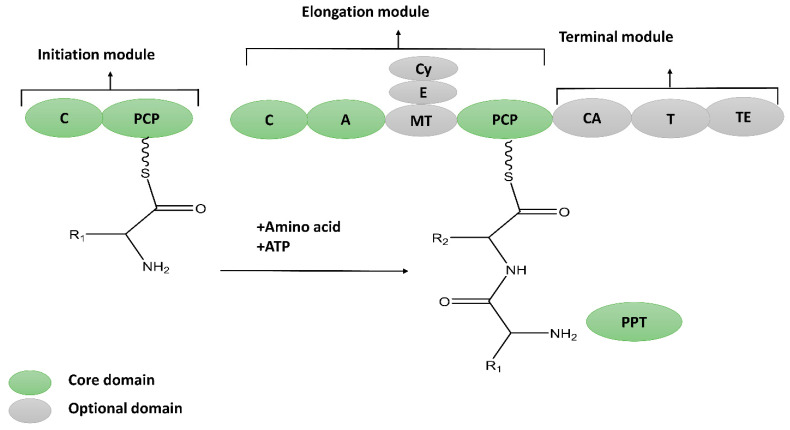
Biosynthesis of non-ribosomal peptides (NRPs), consisting of core and auxiliary domain. Core domain encoding NRP includes A; Adenylation, PCP; peptidyl carrier domain, C; condensation ans TE; thioesterase domain. Auxiliary or optional domains responsible for cyclization (Cy), N-methylation (MT) and epimerization (E).

NRPs are molecular assembly machines that use multi-modular enzyme complexes rather than a DNA template to construct a protein [16]. A module is a part of the non-ribosomal peptide synthetases (NRPS) enzymes that assimilates the amino acid into a peptide backbone of a specific kind. Further, each module may be divided into three domains, adenylation (A), thiolation (T) or peptidyl carrier protein (PCP), as well as a condensation (C) domain that catalyzes the individual steps of NRP synthesis [17] (Figure 2). The synthesis of the primary product might be post-synthetically modified to obtain its mature form via glycosylation, methylation, hydroxylation, acylation, heterocyclic ring formation, and halogenation [18,19]. NRPs exhibit vast structural diversity and may be found as linear, branched, and cyclic structures. So far (January 2022), the Norine (Non-ribosomal peptide database) contains 1740 peptides, including information about their biosynthesis, structures, and evolution [20].

The NRPs may be synthesized via a multi-enzyme thio-template mechanism or without the multi-enzyme [21]. The NRPs synthesized through a thio-template are usually 2–50 amino acids and some other moieties such as a fatty acid chain, whereas the NRPs synthesized without a multi-enzyme template are comparatively smaller [22]. Based on biosynthetic pathways, NRPs can be divided into thio-template and non-thiotemplate NRPs.

### 2.1. Thio-Template NRPs

The thio-template NRPs can be classified as cyclic lipopeptides and non-cyclic or linear lipopeptides based on their chemical structures (Supplementary Material Table S1).

#### 2.1.1. Cyclic Lipopeptides

Thio-template non-ribosomal cyclic lipopeptides were first isolated during the 1950s–1960s from *Bacillus* spp. The cyclic lipopeptides are primarily synthesized via the sequential addition of residues, either in an iterative or a non-iterative manner. The lipopeptide synthesis pathways are very flexible; therefore, the synthesized peptides are incredibly diverse in nature. The cyclic lipopeptides produced by *B. subtilis* can be classified into four classes [23].

**Figure 3:**
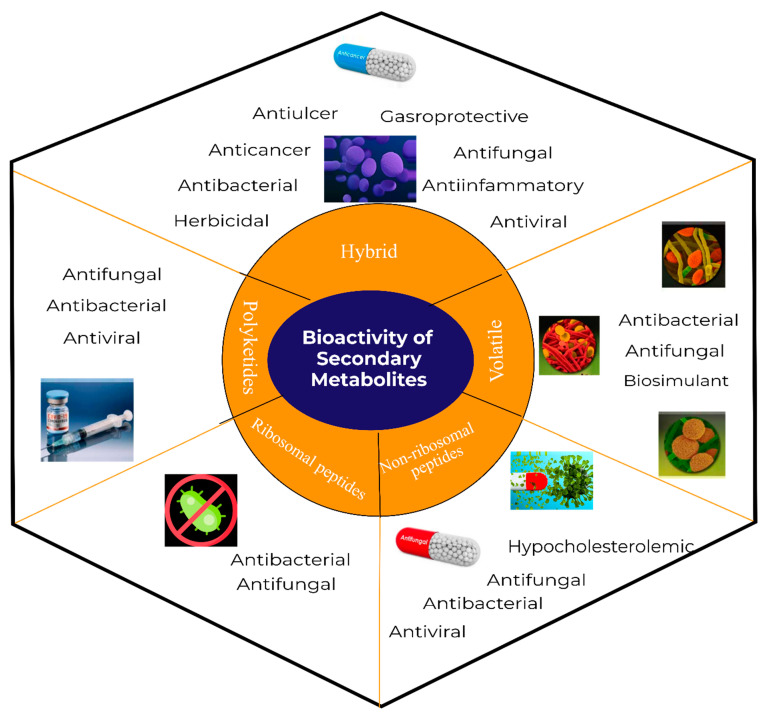
Potential application of secondary metabolites synthesized by *Bacillus subtilis*.

- Surfactins: Surfactin was first isolated in 1968 from the culture supernatant of *B. subtilis*, which exhibited an excellent biosurfactant activity [24]. Subsequently, surfactin was demonstrated to be an antitumor, antibacterial, anticoagulant, and hypocholesterolemic agent [25] as shown in Figure 3. A typical structure of a surfactin is shown in Supplementary Material Figure S1.
- Iturins: In 1949, Walton and Woodruff isolated the first antifungal iturin from *B. subtilis*. Later on, in 1950, a second similar compound iturin was reported, whose name was derived from Ituri (the name of the place in Congo where the soil sample was collected) [26]. The exact structure of iturin was elucidated to be a cyclic hepatolipopeptide attached to the alkyl chain (Figure S1). Iturins are known to display potent antifungal activity and could be used as an active ingredient in several phytopathogen control products. The closely related cyclic lipopeptides could be classified as iturin: bacillomycin L [27], mycosubtilin [28], bacillomycin D, bacillomycin F [29], mojavensin A [30], and subtulene A [31].
- Fengycins: In 1986, Japanese and German scientists simultaneously discovered fengycin from *B. subtilis* [32]. Initially, it was determined that fengycin inhibits the growth of filamentous fungi and is ineffective against non-filamentous fungi and bacteria. Later on, however, its antiviral [10], antibacterial [33], and anticancer properties were reported [34]. It also exhibited a plant growth-promoting property, which is desirable in the agriculture industry.
- Kurstakins: Kurstakin is a lipo-heptapeptide exhibiting antifungal activity produced by several *B. subtilis* strains. Kurstakins cannot be recovered from the culture supernatant but are found in association with the producing cells [35]. Nevertheless, the co-infection study conducted with the producing and non-producing strains demonstrated that it is extracellular [36]. This contradiction suggests that kurstakin is an extracellular metabolite having a high affinity to the cell membrane. This affinity is probably due to the presence of histidine, which gives a positive charge to kurstakin and allows its electrostatic interaction with the phospholipid of a membrane.
- Plipastatins: Plipastatin was first reported from *B. cereus* as an antiphospholipase, before being identified in *B. subtilis* [37]. Plipastatins are closely related to fengycin. The alteration happens from the inversion of two stereocenters, offering a distinct 3D structure to plipastatin’s backbone. Notably, these apparent small structural differences result in a loss of antifungal activity [38].

#### 2.1.2. Linear Lipopeptides

Linear lipopeptides have been produced by several *B. subtilis* strains. They can be classified into the following two subclasses

a. Gageopeptides

Recently, several linear lipopeptides have been reported and characterized from *B. Subtilis*. For instance, Gageostatin was reported from a marine-derived *B. subtilis* [39]. Gageostatin consists of 3-beta hydroxyl fatty acid attached to heptapeptide (Figure S2). It is composed of the same residues as reported for surfactin. However, differences were found in their structures and molecular masses. Gageostatins were found in linear form with exclusively L-leucine, while surfactins are cyclic lipopeptides with L and D-leucine.

Interestingly, it was reported that a mixture of the two metabolites (gageostatin A and gageostatin B) was more effective and appeared to work synergistically against bacteria and fungi. However, the future application of gageostatins as a drug candidate may be limited due to their broad cytotoxicity [39]. Gageotetrins A–C and gageopeptides A–D are linear antimicrobial peptides comparable to gageostatins. Unlike gageostatins, they exhibit no cytotoxicity and strongly antagonize bacteria and fungi [40]. They are probably synthesized via a hybrid biosynthesis pathway, and gageotetrin A is a potential precursor for gageotetrin B. Gageotetrins were more recently isolated from *B. subtilis* strain 109GGC020 and displayed promising antibacterial and antifungal activities in a time-dependent manner [40].

b. Siderophores

Siderophores are small molecules having a high affinity toward ferric iron. Besides iron scavenging, they are also used to form stable complexes with environmentally important metals. Based on their chemical moiety, siderophore can be categorized into three types, i.e., hydroxamate, catecholate, and carboxylate siderophores. Most of the siderophores produced by bacteria are catecholate, and few are carboxylate and hydroxamate [41]. Bacillibactin is a well-known catecholate siderophore produced by different *B. subtilis* strains that exhibits strong antibacterial properties and moderate cytotoxicity [42]. The chemical structures of representative siderophores are shown in Figure S2. *Bacillus* spp. are widely studied for the synthesis of bioactive metabolites. However, their siderophore-producing capabilities have not yet been much explored. Previously, a siderophore-producing *Bacillus* spp. was reported that enhanced the bioremediation of metals and increased plant growth. *B. subtilis* strain CAS15 was isolated from the rhizosphere and identified as producing siderophore, and it also inhibits the growth of phytopathogens [43]. Similarly, the siderophore-mediated bioaccumulation of cadmium (Cd) has been reported in *B. subtilis* and demonstrated as a substitute bioremediation strategy [44]. In recent years, the biological control of phytopathogens has been the subject of research, because it helps in limiting the use of hazardous chemically synthesized pesticides. The siderophore-producing bacteria provide a promising alternative disease management strategy, as they are able to enhance crop yields and, at the same time, protect plants from pathogens.

### 2.2. Non-Thio-Template NRPs

*B. subtilis* is capable of synthesizing antimicrobial NRPs via non-thio-template mechanism. Rhizocticins are non-thio-template peptides that consist of an arginine linked with L-2-amino-5-phosphono-3-*cis*-pentanoic acid. Sometimes they are supplemented with leucine, isoleucine, and valine [45]. Besides rhizocticins, *B. subtilis* can also synthesize dipeptide NRPs such as bacilysin (tetain) and chlorotetain (Supplementary Material Figure S2). Regardless of their simple composition (L-alanine link with L-anticapsine), they exhibit strong antibacterial and antifungal activity [46]. The antibacterial activity is mediated by the L-anticapsine, which inhibits the synthesis of glucosamine-6 phosphate. Its inhibition stops the synthesis of peptidoglycan, which is a major constituent of the bacterial cell wall. For antifungal activity, it has been suggested that anticapsine can suppress the biosynthesis of chitin and fungal cell membrane mannoproteins [47]. Tetain and chlorotetain are shown to inhibit the growth of *Aspergillus fumigatus* and *Candida albicans* [48]. Mycobacillin and bacitracin are non-thio-template polypeptides produced by *B. subtilis*. Mycobacillin inhibit the growth of *Aspergillus niger* by altering its cell membrane [49]. The biosynthesis of mycobacillin is unique. The NRPS complex catalyzed and divided it into three parts, i.e., A, B, and C, but it does not use the thio-template mechanism [50]. Each part of the complex comprises a single polypeptide enzyme that catalyzes the polymerization of pentapeptide A, nonapeptide B, and tridecapeptide C. Bacitracin is a heptapeptide that widely antagonizes Gram-positive bacteria by inhibiting the biosynthesis of petidoglycan [51].

## 3. Ribosomal Peptides (RPs)

**Figure 4:**
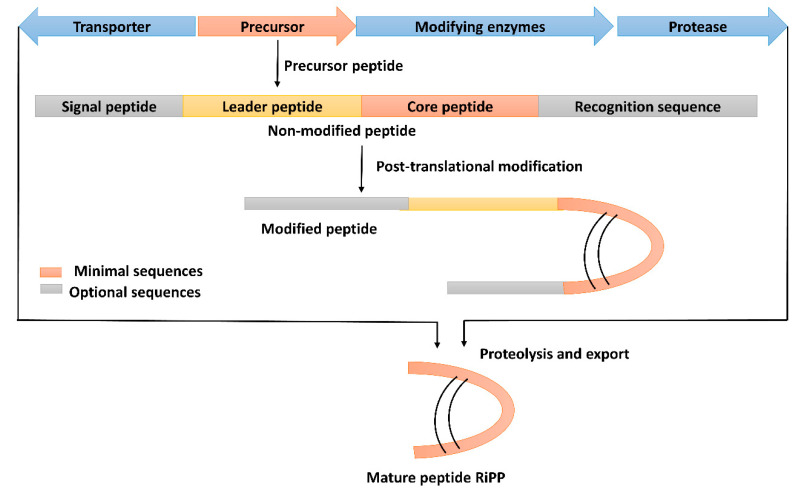
Schematic representation of biosynthesis of ribosomal peptides (RPs). The short precursor peptides are converted into core peptides, and then the core peptide is matured via post-translational modifications.

Ribosomal peptides (RPs), also known as ribosomally synthesized and post-translationally modified peptides (RiPPs), are derived from a relatively short precursor peptide and are matured through post-translational modification [52]. Several enzymes are involved in these modifications; thus, structurally diverse peptides are generated (Figure 4). In order to classify the RiPPs produced from *B. subtilis*, several classifications have been proposed, and the classification based on the biosynthetic pathway or chemical structure is reasonable, as reported for bacteriocin produced by *Enterococcus* spp. and *Streptococcus* spp. [13]. Consequently, the known *B. subtilis* producing RiPPs could be classified into three main classes and several subclasses (Supplementary Material Table S2).

### 3.1. Class I—RiPPs

Class I consists of biologically active short peptides that are ribosomally synthesized and undergo post-translation modification (PTM), resulting in a unique structure and properties. According to PTM differences, class I can be divided into several subclasses.

#### 3.1.1. Lanthipeptides

Class 1 lanthipeptides consist of highly diverse post-translationally modified peptides that characteristically contain cross-link thioether between non-proteinogenic lanthionine and 3-methyllanthionine [53]. Lanthipeptides are produced as precursor peptides and consist of a leader and a core peptide. The precursor peptide is post-translationally modified and cross-linked with thioether, and, subsequently, the leader peptide is removed, and a mature lanthipeptide is released [54]. Lanthipeptides exhibit promising antimicrobial activity, and, indeed, a lanthipeptide “nisin” is commercially used in the food industries. Nisin prevents the synthesis of peptidoglycan transglycosylation and forms a membrane-spanning pore [55]. Several gene clusters are associated with the biosynthesis and maturation of class I lanthipeptides, as identified in the *B. subtilis* genome using computational tools. Recently, a more robust computational tool has been designed to identify the lanthipeptide gene cluster from genomic data [56]. However, their specific products are yet not purified from the producer strains.

#### 3.1.2. Lasso Peptides

Lasso peptides are a relatively newly characterized class of RiPPs composed of short-chain peptides comprising an N-terminal macrolactam by which the C-terminal is linked [57]. The N-terminal “ring” is formed by 7 to 9 amino acids that are linked by an isopeptide bond between the N-terminal amino group of the first residue and the carboxylate chain of glutamate or aspartate residue [54]. The first residue of lasso peptides, cysteine or glycine, is highly conserved. Therefore, bioinformatics approaches are recommended for the discovery of new lasso peptides [58]. However, few lasso peptides have been discovered with alanine or serine as their first amino acid [59]. Lasso peptides’ gene clusters have been identified from the *B. subtilis* genome, but the specific product is yet not fully characterized. Lasso peptides are important antimicrobial peptides, and their biosynthesis involves the synthesis of a precursor A-peptide via A-protein. The precursor is post-translationally modified via B-protein and C-protein. B-protein is an ATP-dependent lasso protease that removes the leader peptide, and C-protein is an ATP-dependent synthetase (lasso cyclase) that catalyzes the synthesis of the macrolactam ring between the N-terminal of an amino group and the side chain of glutamate or aspartate in the peptide. Due to the specific threaded loop topology, the peptides resemble “lassos”, hence the term “lasso peptides” [54].

#### 3.1.3. Sactipeptides

Sectippetides are biologically active peptides with exceptional cross-links between the sulfur of cysteine and the alpha carbon of other residues catalyzed by S-adenosylmethionine (SAM) [60]. The post-translational link of a thiol group to the alpha carbon is unusual in RiPPs and is responsible for the antimicrobial activity of sactipeptides [61,62]. Genome mining can efficiently detect the unique SAM enzyme whose coding genes are co-localized with the sactipeptides biosynthesis gene cluster [63]. Several sactipeptides have been identified from *Bacillus* spp., such as subtilosin A, a sactipeptide produced by the synthesis of *B. subtilis* 168 from the predecessor by the proteolytic cleavage of the leader peptide and the cyclization of the N and C-terminals via covalent linkage [64,65]. Further, modification of threonine, cysteine, and phenylalanine occurs, and the mature peptide is exported via ABC transporters [13]. Sactipeptides are reported to be active against *Bacillus* spp. *Listeria monocytogen*, *Gardnerella vaginalis*, and *Enterococcus faecalis* by making pores in their cell membrane [65,66,67].

#### 3.1.4. Linear Azole-Containing Peptides (LAPs)

The linear azole-containing peptides (LAPs) are heterocyclic peptides derived from the threonine, cysteine, and serine of a short precursor peptide [68]. They consist of four obligatory modules: a precursor peptide that is also known as peptide A, a heterotrimeric enzyme complex (dehydrogenase B), and cyclodehydratase C and D. Plantazolicin is a LAP produced by *B. amyloliquefaciens*. Its biosynthesis initiates with the formation of azoline heterocycles via the C and D complex from threonine/serine and cysteine, followed by dehydrogenation by B leading to the synthesis of the corresponding azole [69,70]. Regardless of the low amino acid identity of the BCD complex between LAP loci, numerous reports revealed that BCD genes could be associated with various LAP biosynthesis pathways by converting the precursor peptide into the active RiPP [70,71]. Therefore, these genes are used in genome mining [72]. Sonorensin was initially reported from a marine-derived *Bacillus* sp. that effectively antagonizes the growth of both Gram-positive and negative bacteria. Further, it was proposed that growth inhibition occurred due to the increased cell membrane permeability. The sonorensin-coated low-density polyethylene film efficiently controls the food spoilage of Gram-positive bacteria such as *S.aureus* and *L. monocytogenes* [73]. The bio-preservative property of the sonorensin-coated film in meat and vegetables demonstrates its potential application in the food industries.

#### 3.1.5. Thiopeptides

Thiopeptides are an emerging group of antibiotics and include more than 100 compounds. Thiopeptides not only exhibit antibacterial activity but also possess broad bioactivities such as anticancer, antiplasmodial, and anti-immunosuppressive activities [74]. Few completely characterized thiopeptides have been reported from the *B. subtilis* group. Recently, only one thiopeptide was purified and identified among 80 thiopeptide biosynthetic gene clusters detected via genome mining approaches [75].

#### 3.1.6. Cyclic (Head-to-Tail) Peptides

The head-to-tail cyclic peptides are relatively long peptides with 35–70 amino acid residues. The peculiar features of cyclic peptides are not only their large size but also the modifying enzymes associated with their cyclization. Due to their macrocyclization, these peptides are relatively resistant to higher temperatures, pH changes, and several proteases [76]. These peptides are distinguished from lanthipeptides in that they do not contain lanthionine, methyl lanthionine, and hydrated amino acid residues [77]. Amylocyclicin was reported as a new head-to-tail cyclized peptide from *B. amyloliquefaciens*, which is derived from 112 amino acid residues precursor encoded by the *acnA* gene [78]. The cyclization of amylocyclicin occurs between leucine one and tryptophan 64. Amylocyclicin inhibits the growth of Gram-positive bacteria, including *B. subtilis* [78]. Recently, another novel cyclized peptide, “pumilarin”, was detected via genome mining in the *B. pumilus* genome and was reported to have a cyclized structure and exhibit a wide range of bioactivities [79]. Pumilarin was found to be post-translationally modified so that its N and C-terminals are linked via a peptide bond. The pumilarin biosynthetic gene cluster comprises the *pumA*, *pumB*, *pumC*, *pumC1*, and *pumD* genes [79]. However, the exact biosynthetic pathway for pumilarin is yet to be elucidated.

### 3.2. Class II Peptides

Class II metabolites are heat stable (121 °C), short peptides of less than 10 kDa, and are usually not post-translationally modified [80,81]. Previously, lactic acid bacteria were reported to be the main producer of RiPPs due to their long history of safe use in food [82]. In 1960, nisin was approved as a safe food additive and used in more than 50 countries as an antibacterial agent against Gram-positive bacteria [83]. Nevertheless, the quest for new antimicrobial agents rapidly stretched to other RiPP producing bacterial species. Bacillus species have become increasingly attractive due to being “generally recognized as safe (GRAS)” and having a broader antimicrobial spectrum. [21,84,85].

The non-modified RiPPs produced by the *B. subtilis* group can be further divided into three subclasses based on the conserved amino acid motifs near N-terminus.

#### 3.2.1. Pediocin-like Peptides

The pediocin-like peptides inhibit numerous clinically relevant pathogens and have a conserved YGNGVXC motif. Despite their great potential as antibacterial agents, the problems associated with their commercial-scale production limit their industrial application. So far, coagulin is the single complete characterized pediocine-like peptide reported from *B. coagulans* [86]. Coaguline was first reported in 1998, and the complete amino acid sequence was reported in 2000 [80].

#### 3.2.2. Other Non-Modified Peptides

The non-modified peptides have a conserved motif of DWTXWSXL at the N-terminus. In 2001, a hydrophobic and thermotolerant antimicrobial peptide, lichenin, was purified and characterized from *B. licheniformis* culture [87]. Recently, the biosynthetic gene clusters encoding the class IIb non-modified peptide BhIA were reported from the *B. subtilis*, *B. lechniformis*, *B. pumilus*, and *B. amyloliquefaciens* genomes via genome mining approaches. The structural analysis revealed significant similarities with holins produced by *Geobacillus* spp [88]. Holins are phage-encoded peptides involved in the disruption of bacterial cell membranes [89]. However, the function of each biosynthetic coding module remains unknown. The holin-like BhIA metabolite exhibited antibacterial activity against pathogenic Gram-positive bacteria such as *Micrococcus luteus* and multi-drug resistant *S. aureus* [90]. The peptide BhIA is composed of 70 amino acids with a transmembrane domain at the N-terminus. Several hydrophilic amino acid residues at the N-terminal and specific membrane topology distinguish BhIA from holin [90]. Aureocin A53 is another new member of the non-modified peptides whose biosynthetic gene cluster was detected in the *B. pumilus* genome. Aureocin antagonizes the growth of *L. monocytogenes* by disturbing the cell membrane and inhibits the synthesis of DNA, protein, and polysaccharides simultaneously [91]. LCI was initially isolated from B. subtilis strain A014 and exhibits promising bioactivity against the plant pathogen *Xanthomonas campestris* [92]. *X. campestris* causes leaf blight disease in rice, which is a severe threat to rice production and causes significant losses in the rice field annually. LCI is a beta-structured antibacterial peptide comprising 47 amino acid residues. It also carries a hydrophobic core consisting of valine, tryptophan, and tyrosine [93]. LCI is a cationic peptide causing short-lived channels in the target bacterial cell membrane [93].

#### 3.2.3. Large Antimicrobial Peptides

The large antimicrobial peptides are relatively larger and include bioactive metabolites having a size of more than 10 kDa. Their biosynthetic gene cluster consists of an immunity gene and a structural gene. Several *Bacillus* species, such as *B. thuringiensis*, *B. coagulans*, and *B. cereus*, are reported to produce large antimicrobial peptides [88]. However, as yet, none have been reported from the *B. subtilis* group.

## 4. Polyketides (PKs)

**Figure 5:**
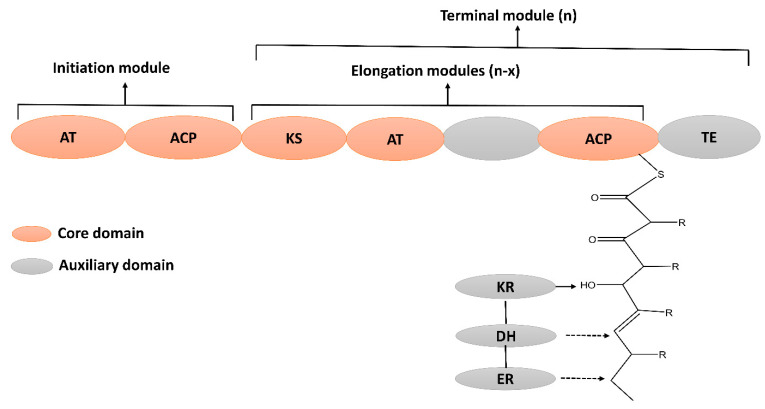
Schematic representation of enzyme domains involved in the biosynthesis of Polyketide (PKs). Core and auxillary (optional) domains are color coded. Core domains: AT; Acyltransferse, ACP; Acyle carrier protein, KS; Ketosynthase and TE; Thioesterase. Auxiliary domains: KR; Ketoreductase, DH; dehydrase, and ER; Enoyl acyle reductase.

Polyketides are structurally diverse bioactive metabolites that contain an alternative methylene and carbonyl group [94]. Polyketides are widely used as therapeutic agents to treat numerous diseases [95]. For instance, tetracycline and erythromycin are used as antibacterials [96], amphotericin is used as an antifungal [97], and anthracyclin is used as an antitumor drug [98]. The biosynthesis of PKs is carried out by a multi-domain enzyme, which consists of ketosynthase, acyltransferase, and thioesterase. Its biosynthesis is initiated by loading the acyl CoA on acyl carrier protein (ACP), catalyzed by acyltransferase (AT). The ketosynthase (KS) extends the carbon chain via decarboxylative condensation. The ketoreductase (KR), enoyl reductase (ER), and dehydratase (DH) domain may further modify the beta-keto group to produce diverse PKs (Figure 5). Subsequently, the thioesterase domain terminates the elongation process by cyclizing or hydrolyzing the PK chain from ACP and releasing a mature PK peptide [99].

PKs can be classified into three subclasses based on the structural organization of the functional module, i.e., type I PKs, type II PKs, and type III PKs. The type I PKs consist of a large multi-functional enzyme complex carrying several modules bonded covalently and linearly arranged. Type II PKs are multi-enzyme complexes that consist of individual monofunctional enzymes combined during the biosynthesis of ketides. In contrast, type III PKs are chalcone synthase (CHS) like polyketide synthetases, which activate the CoA thioesters directly without the ACP domain [100]. Apart from the structural organization of the functional domain, PKs can be categorized as iterative and non-iterative depending on the number of ketosynthase involved in the biosynthesis of PKs. Bacteria used non-iterative type I polyketide synthase (PKS) enzymes to produce polyketides, and this consensus linearity is employed to identify PKs via genome mining approaches [101]. Besides these differences, due to the great diversity, some other alteration has also been observed. As some times PKs biosynthesis pathways mixed by combining different types of PKSs or even can be associated with NRPSs or fatty acid synthetases to produce a hybrid peptide such as compactin, bacillaene, and fusarin *C. bacillaene* was previously classified as a PKs-polyene [22]. But, here, based on biosynthetic pathways classified as hybrid PKS/NRPS and will discuss later in hybrid metabolites. PKs can be divided into several classes based on typical structure and carbon skeleton [102]. However, the PKs produced by *B. subtilis* can be divided into two major types, i.e., Polyenes and enediynes (Supplementary Material Table S3).

### 4.1. Polyenes

a. Difficidin

Difficidins are unsaturated macrocyclic polyene synthesized by the type 1 PKS (Supplementary Material Figure S3A). Oxydifficidin is an oxidative form of difficidin having an additional hydroxyl group at position 5 [103]. It is encoded by the *diff* operon that has 14 open reading frames. Several KR, DH, and ER domains are missing within the *diff* operon and deviate from the colinearity rule. Moreover, the function of *diffJ* and *diffK* are unknown, and their activities do not appear in the final product [104]. Difficidin has broad-spectrum antibacterial activity and inhibits the biosynthesis of protein in *E. coli* [103,105]. Difficidin is produced by *B. amyloliquefaciens* ATCC strain numbers 39,320 and 39,374 (classified initially as *B. subtilis*) [103]. *B. amyloliquefaciens* strain FZB42 showed biocontrol activity against *Xanthomonas oryzae* by producing difficidin. Scan electron microscopy results showed that difficidin inhibits the growth of a phytopathogen by rupturing the targeted bacterial cell wall. Further, the biocontrol experiments revealed that difficidin caused the downregulation of genes associated with Xanthomonas cell wall synthesis, cell proliferation and virulence [106]. Collectively these studies discuss the future prospective of the strains as a biological control agent against plant pathogens.

b. Aurantinin

The antibacterial exhibiting polyketide aurantinin A and B were isolated initially from Bacillus aurantinus [107]. Recently, along with these two aurantinins, C and D were reisolated in combination with the genome mining approach [108]. The structure of aurantinin and its analogues are very unusual, as they have 5, 6, 7, and 8-membered rings with a highly diverse tail (Supplementary Material Figure S3B). Nevertheless, the absolute structure is yet not elucidated, leaving numerous questions about conformation unanswered. Aurantinin B, C and D exhibit promising antibacterial activity against *Clostridium sporogenes* and *S. aureus*. However, they did not show any cytotoxicity against human epithelial colorectal adenocarcinoma and cellosaurus cell lines. The antibacterial mechanism was examined and revealed that aurantinin B-D disrupts bacterial cell membranes causes breakage of cytoplasm and leads to cell death [108]. Owing to their safety profile and discriminatory activity against G-negative bacteria highlights its scope for commercial applications.

Besides that, four antibacterial metabolites have recently been isolated from marine *B. subtilis* that exhibit antagonistic activity against G-negative food pathogens [109]. Their biosynthetic pathway was speculated to be synthesized via the PKS. Their cytotoxicity, antifungal activity, and complete structure are still unclear.

c. Macrolactins

Macrolactins and their derivatives (7-*O*-succinyl or 7-*O*-malonyl) are synthesized via a type 1 PKS. Macrolactins inhibit the growth of bacteria and have been isolated from various species such as *Bacillus sp.* strain AH1591-1 and *B. amyloliquefeciens* strain FBZ42 [110,111]. Macrolactins usually consist of 24 lactone rings and three diene moieties in the carbon skeleton. Its biosynthetic gene cluster mln composed of nine operons and 11 KS domains with acetate and malonate is the only used building block. Like *dif* gene cluster organization, *mln* appears in an occasional splitting of the modules. The second module is split between *mlnB* and *mlnC*, and a similar arrangement can be observed for modules number 5, 7, 8, and 10. A comparison of catalytic domain organization revealed that the ER domain is missing in module number 2, while two DH domains are missing in modules 7 and 10 [110].

The *B. subtilis* group mostly produces biosynthetic derivatives of macrolactin A (Supplementary Material Figure S3C). For instance, *7-O* succinylmacrolactin A showed antibacterial activity against *S. aureus* with mild cytotoxicity [112,113]. It also exhibits anticancer activity via the CYP2P9 and P13K pathways [114,115,116]. Conveniently, *7-O* succinylmacrolactin A also shows anti-inflammatory activity via the same pathways [112]. Compared to macrolactine A, *7-O* succinylmacrolactin A might be a better candidate as a lead due to its superior stability and pharmacokinetics [115,117]. The compound *7-O*-malonylmacrolactin A exhibits similar anti-inflammatory activity to macrolactin A and *7-O* succinylmacrolactin A; however, due to its activity against MRSA, it gained more attention [112]. Bacillomycin D and *7-O* malonylmacrolactin A produced by the *B. subtilis* group have been employed as biocontrol agents against phytopathogenic bacteria and fungi with bioorganic fertilizers [118,119]. Therefore, the coproduction of various bioactive metabolites might be a productive approach for wide-ranging biological control, highlighting the value of *B. subtilis*.

Moreover, aromatic and unsaturated macrolactin have been reported with *7-O*-6′-(2″-acetylphenyl)-5′-hydroxyhexanoate-macrolactin A, demonstrating a fine example of esterification of two PKS products [113]. The compound *7-O-6*′-(2″-acetylphenyl)-5′-hydroxyhexanoate-macrolactin A was isolated from a seaweed associated *B. subtilis* with good antibacterial activity against Gram-positive bacteria [113]. However, its structure is yet to be elucidated. Conversely, *7-O-2*′E-butenoylmacrolactin A was extracted from sea-sediment-derived *B. subtilis* and showed moderate antifungal activity against *Colletotrichum gloeosporioides* and *Pestalotiopsis theae* [116]. The compound *7-O*-methyl-5′-hydroxy-3′-heptenoate macrolactin A isolated from the algal-associated *B. subtilis* strain MTCC 10,403 displayed moderate antibacterial activity [120], but the investigator further did not report bioactivity and structure-related information.

Macrolactin B is *7-O-*β-glycosylate was first isolated in 1989 and was reisolated from marine B. subtilis [121,122]. Macrolactin B exhibit potent antifungal activity; however, unlike macrolactin A, it is not cytotoxic, indicating the structure-activity relationship of macrolactins. Macrolactin W is the only example of macrolactin which is both *7-O* glycosylated and esterified. Its antimicrobial property is similar to macrolactin A and B, though it has no cytotoxic activity [122,123]. The cytotoxic, antiviral, and anti-inflammatory activity is observed for the parent metabolite macrolactin A and as yet not determined for its derivatives. It is anticipated that these compounds are hydrolyzed in vivo and release the active metabolite macrolactin A.

### 4.2. Enediynes

To date, the PK enediyne is the most cytotoxic natural product, and its application as an anticancer drug has been clinically demonstrated [124]. Due to the substantial cytotoxicity of enediyne, its application is minimal. However, its application in various antibody-drug conjugates and polymer-based drug delivery systems has had great success [125,126]. Enediynes are commonly produced by *Streptomyces* spp. However, recently reported from *Bacillus* sp. via the genome mining approach [127]. The complete biosynthetic machinery and structure yet remain to be elucidated.

## 5. Hybrid Metabolites

Hybrid metabolites are the products of biosynthetic pathways that comprise both (NRPS/PKS) types of modular enzymes. Questions relating to the synthesis of hybrid products are of great present-day interest, as their answer concerns genetic engineering efforts. Both depend on thio-template for acyl chain elongation and monomers triggering. Presently, the factors of molecular events that offer a hybrid pathway to accommodate various assembly line chemical moieties functionally are yet not completely elucidated. However, based on the current knowledge, the hybrid metabolites produced by *B. subtilis* can be classified as bacillaene and isocoumarins (Supplementary Material Table S4).

a. *Bacillaene*

**Figure 6:**
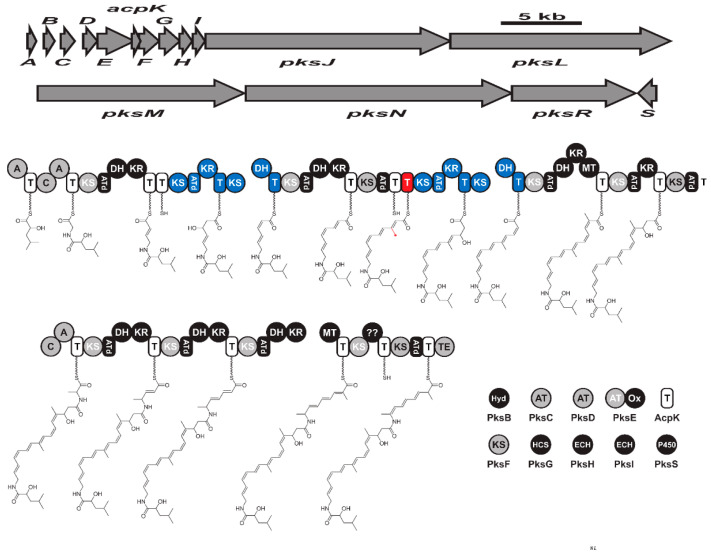
The biosynthetic gene cluster (pksX) and biosynthesis of hybrid bacillaene. Key: KS (ketosynthase), AT (acyltransferase), T (thiolation), DH (dehydratase), KR (ketoreductase), MT (methyltransferase), A (adenylation), C (condensation), ATd (AT-docking), Hyd (Zn-dependent hydrolase), Ox (flavin mononucleotide-dependent oxidase), HCF (HMG-CoA synthase), ECH (enoyl-CoA hydratase), and TE: (thioesterase).

Bacillaene has a linear structure (Supplementary Material Figure S3D) and was first reported from *B. subtilis* strains 55,422 and 3610 [128]. It is encoded by a hybrid PKS-NRPS biosynthetic gene cluster known as bacillaene PksX synthase (Figure 6). The *pksX* mega gene cluster in *B. subtilis* 168 genome consisted of 5 open reading frames named *pksJ*, *pksL*, *pksM*, *pksN*, and *pksR* [104,129]. The first two adenylation domains of *pksJ* incorporate glycin and α-hydroxy-isocaproic acid. The third adenylation domain (*pksN*) is responsible for the incorporation of alanine. While the three open reading frames *pksC*, *baeD*, and *baeE* encode for three separate AT domains are responsible for incorporating malonyl-CoA [130]. Bacillaene inhibits the growth of various bacteria and fungi such as *Myxococcus xanthus*, and *Trichoderma* spp. [131,132]. Bacillaene selectively inhibits the biosynthesis of protein in bacteria, indicating a selective inhibition of other strains in their habitat [128].

b. *Isocoumarins*

Isocoumarins form a large, diverse class of biologically active metabolites with more than 200 metabolites [133]. However, fewer are reported from *B. subtilis*. It has been reported that *B. subtilis* specifically produce isopropyl-8-hydroxy-3,4-dihydroisocoumarins with an active side chain or functionalized amino acid [134]. Amicoumacin A-C (Supplementary Material Figure S3E,F) and amicoumacin F are dihydroisocoumarins initially reported from *B. pumilus* [135,136,137]. Later, they were reported from *B. subtilis* and determined its strong antibacterial activity against the gastric pathogen *Helicobacter pylori* [138]. Biosynthesis of these bioactive metabolites has been recently reported from *B. subtilis* 1779 via a genome mining approach [139]. The BGC encoding amicoumacin was predicted to be 47.4 kb in size and consists of 12 open reading frames. Further, the investigator expressed the biosynthetic gene cluster in a heterologous host and obtained amicoumacin A–C. Based on these findings, they predicted the biosynthetic pathway for amicoumacin to be synthesized via hybrid PKS-NRPS modular enzymes [139]. The biosynthetic gene cluster consists of eight modules that synthesize two discrete pre-amicoumcin molecules. The unique dihydroisocoumarins core is likely to be synthesized via removing the AmiJ-M megasynthase complex to produce an oxygenated PK chain that reorganizes into a cyclic dihydroisocoumarin [139]. Amicoumacin exhibit promising anti-inflammatory and antibacterial activity and has gained increased interest as a pro-drug candidate. Specifically, amicoumacin A is more attractive due to its potent anticancer activity and antibacterial activity against MRSA, with MIC less than 1 µg/mL [136,137]. The antibacterial mechanism of amicoumacin was recently shown to be bind on the ribosome and inhibit protein biosynthesis [140,141]. Amicoumacin A and C exhibit antibacterial activity, while amicoumacin B is non-antibacterial. Interestingly, the hydroxy group in amicoumacin A and C is responsible for their antibacterial activity as the phosphate ester-containing amicoumacin B lacks antibacterial activity [142].

Bacilosarcin A (3-oxa-6,9-diazabicyclo [3.3.1]nonane ring) and B (rare 2-hydroxymorpholine moiety) with unique heterocyclic core structures were isolated from the marine-derived *B. subtilis* strain TP-B0611 [143]. Later on, their complete chemical structures and structure-activity relationships were demonstrated [144]. Unlike amicoumacin A and B, bacilosarcin A and B displayed marginal anti-herbicidal activity. The low anti-herbicidal activity reflects the side chain functional group and highlights the importance of the side chain for bioactivity. This hypothesis is further supported by the non-antibacterial activity of bacilosarcin C, which has –COOH (carboxylic) instead of –NH_2_ (amide) group [145].

The piperazin-containing damxungmacin A (dihydroisocoumarin) has an unusual heterocyclic ring that displays weak antibacterial activity and cytotoxicity [146]. Unlike other dihydroisocoumarin, damxungmacin exhibits weak bioactivity, low yield, and a complex molecular structure that offers distinctive bioprocessing techniques development. Similarly, damxungmacin B was isolated and showed no bioactivity against tested strains.

Hetiamacin A has shown promising antibacterial activity against oxacillin resistant *S. aureus*, *Streptococcus pneumonia*, and *S. epidermidis* with MIC less than 2 µm/mL [147,148]. The small differences in their structure and vast differences in their biological activity make them an attractive area of research to investigate the structure-activity relationship. Similar to amicoumacins, hetiamacin B has shown potent antibacterial activity; however, the structure configuration and biosynthesis of hetiamacin is still unknown. Hetiamacin C and D were also isolated from the same bacillus strain, but due to the low yield, their biological activity was not determined [149].

The instantaneous application of amicoumacin in agriculture and human medicine may not be appropriate. However, their strong anti-MRSA activity appears an attractive starting point for drug development. To date, their mode of action is yet not fully elucidated, therefore, compelling further investigation. Whereas some isocoumarins discussed above have already been synthesized, and the paths could be further extended towards sustainable and economical production, amicoumacin derivatives demand further studies to demonstrate the structure-activity relationship of the metabolites.

## 6. Volatile Metabolites

Volatile metabolites enhance the efficacy of several secondary metabolites produced by bacteria in a specific habitat. The increased vapor pressure facilitates the low molecular biologically active metabolites to move over a longer distance and act on the target organism in soil [150,151]. *B. subtilis* emit highly diverse volatile secondary metabolites, including terpenes, nitrogen, and sulfur-containing compounds, benzenoids, and hydrocarbons [152]. These metabolites are primarily considered cell signaling molecules mediating inter and intracellular interaction [153]; however, they also displayed antifungal and antibacterial activity [154,155]. For instance, a volatile dimethyl disulfide, promote the growth and survival of plants along with antimicrobial activity [156]. *B. subtilis* releases many volatile metabolites with an average of 14 metabolites per strain [12]. These volatile metabolites are involved in the biogeochemical cycles and bioconversion of the food chain and numerous metabolic activities such as nitrification and nitrogen mineralization. *B. subtilis* producing volatile metabolites may be inorganic or organic volatiles (Supplementary Material Table S5).

### 6.1. Volatile Inorganic Metabolites

The volatile inorganic metabolites are a by-product of the primary metabolites. They are usually nitrogen and sulfur-containing compounds such as ammonia (NH_3_), hydrogen sulfide (H_2_S), and hydrogen cyanide (HCN) (Supplementary Material Figure S4). Nitrogen-containing compounds are mainly emitted in the top sediment layer by denitrifying *B. subtilis* strains. The *B. subtilis* group also produces nitric oxide, which induces a systemic acquired resistance in plants against *Ralstonia solanacearum* [157]. In an oxygen-deficient environment, bacteria emit various volatile inorganic metabolites such as hydrogen sulfide and hydrogen. These compounds act as a precursor for amino acid, antimicrobial metabolites synthesis, or act as electron acceptors. *B. subtilis* produce hydrogen sulfide from sulfate hydrolysis or a by-product of L-cysteine and L-methionine catabolism [158,159]. It is reported that hydrogen sulfide inhibits the growth of phytopathogens, such as *Penicillium italicum* and *Aspergillus niger* [160]. Indeed it is also demonstrated that it provides self-protection to producer organisms against antibiotics [161]. Hydrogen cyanide produced from amino acid (glycine) catabolism potentially inhibits the growth of plant pathogens such as *Agrobacterium tumefaciens* crown and *Meloidogyne incognita* juveniles [162].

### 6.2. Volatile Organic Metabolites

Volatile organic metabolites are low molecular weight molecules having a lipophilic moiety, a low boiling point, and high vapor pressure. These characteristics offer fast evaporation and distant distribution in a complex matter like soil [163]. Their production by soil bacteria and distribution in the soil is strongly influenced by the availability of nutrients and oxygen, pH, temperature, soil humidity, architecture, and texture [151,164]. Volatile organic metabolites are mainly a product of the glycolysis and citric acid cycle [163,165]. However, they can also be synthesized via different pathways, such as fermentation, amino acid degradation, terpenes biosynthesis, sulfur reduction, and heterotrophic carbon degradation [166]. So far, 1860 microbial volatile organic metabolites emitted by 944 various microbial species are listed in the mVOC (microbial volatile organic compounds) database [167]. It is reported that about 70% of the *Bacillus* volatile organic compounds (bVOCs) in the mVOC database are either fatty acid or nitrogen-containing metabolites. A few representatives of the volatile organic metabolites are shown in Supplementary Material Figure S4. The volatile organic metabolites emitted by the *B. subtilis* group can be classified into seven subclasses.

#### 6.2.1. Terpenes and Terpenoids

Terpenoids, also known as isoprenoids, are widely produced by all living organisms [168]. The end product of the deoxy xylulose phosphate pathway, isopentenyl pyrophosphate (IPP), and their isomer dimethylallyl pyrophosphate (DMAPP) usually act as precursors for terpenoid biosynthesis [158]. However, terpenoids may also be synthesized from isoprene [169]. As previously shown, the isoprene produced by *B. subtilis* is not synthesized via the deoxy-xylulose phosphate pathway. Isoprenoid plays a vital role in physiological functions such as membrane fluidity, electron transport, light-harvesting, and cell signaling [170]. The involvement of terpenoids in cell signaling is particularly important, as it is associated with some mutualistic, antagonistic, and multitrophic interactions [171]. Isoprenoids may potentially be used as a flavor, nutraceuticals, fragrance, and therapeutic agent in malaria and cancer treatment [172]. However, due to ecological constraints, their natural yields are often insufficient. The vast structural diversity of terpenoids led to the discovery of up to 40,000 compounds by a few groups engaged in pharmaceutical industries [173]. Terpenes are mostly known for their antibacterial, antifungal, antinematode, and insecticidal activities [174,175]. Terpenoids (sesquiterpenes) were previously isolated from *B. subtilis* KSM 6–10 culture supernatant. Additionally, the authors revealed its enzymatic biosynthesis from a cyclic 30 carbon precursor by squalene cyclases (*sqhC*) [176]. Besides that, monoterpenes and isoprene isolated from *B. subtilis* strains were shown to inhibit the growth of nematode and cyanobacteria [175,177]. The mode of action of terpenoids is yet not fully elucidated; however, it could be linked to their lipophilic nature, which enables them to disrupt the integrity of the cell membrane [178]. They may be further classified into three subclasses. (i) Monoterpenes (ii) Isoprene and (iii) Sesquiterpenes [163].

#### 6.2.2. Nitrogen-Containing Metabolites

Nitrogen-containing metabolites can be distinguished based on their degree of cyclization. To date, three groups of non-cyclic (amine, amide, and imines) and five groups of cyclic compounds (pyrazines, azole, pyridines, pyrimidines, and pyridazines) have been detected. Pyrazines are broadly represented among mVOCs and are categorized into two subclasses: higher alkylated and lower alkylated pyrazines [158]. These metabolites are characterized by a strong odor, and most of the *B. subtilis* strains isolated from rhizosphere and fermented products have been considered as pyrazine producers [179,180]. The compound 2,5-Dimethyl pyrazine was isolated from *B. subtilis* strain, exhibiting strong antifungal activity and inhibiting *P. chlamydospora* [181]. Pyrazine produced by B. subtilis strains also inhibits the growth of bacteria such as *E. coli*, *S. aureus*, and *P. valgaris* [182]. The *B. subtilis* strains are also able to produce other biologically active terpenoids such as 1H-imidazole, 1-ethyl inhibits the growth of phytopathogens [163,183].

The complete biosynthetic pathways for nitrogen-containing volatile organic metabolites remain unknown. However, it is proposed that two major paths may be followed, i.e., the terpenoids such as pyrazines are synthesized from non-enzymatic amination [158] or synthesized from the intermediate produced during amino acid catabolism [184].

#### 6.2.3. Sulfur-Containing Metabolites

Sulfur-containing volatile organic metabolites are synthesized from two primary sources, i.e. organic or inorganic [158]. These metabolites often originate from the catabolism of amino acids, such as methionine, and sometimes from cysteine. However, the inorganic sulfate and sulfite may also act as a precursor for sulfur metabolites. The *B. subtilis* group produces numerous antifungal and antinematicidal sulfur metabolites such as S-methyl butanethioate, S-methyl thioacetate, 2-methyl disulfide, and 3-methyl trisulfide. Among these, dimethyl disulfide also exhibits antibacterial activity [185,186,187,188]. It is known that dimethyl sulfide disrupts bacterial cell communication by decreasing the sum of acyl-homoserine lactone [189].

#### 6.2.4. Benzenoids

Benzenoids are a diverse subclass often linked with sulfur or/and nitrogen. Most of the benzenoids produced by the *B. subtilis* group displayed antifungal activity, while some inhibited the growth of bacteria and nematodes. Their antimicrobial mode of action is partially characterized. Nevertheless, the fungal and bacterial cell disruption is documented after exposure to bVOCs.

The *B. subtilis* strain CF-3 was evaluated for their volatile organic metabolites potential. The investigator identified a plethora of volatile compounds based on solid headspace microextraction. Among them, benzothiazole exhibiting strong antimicrobial activity against fruit fungal pathogens [190]. The volatile organic compounds produced by *B. amyloliqueficiens* were evaluated for their effect on the growth and pathogenicity of tomato bacterial pathogen *R. solanacearum*. The results showed that the strain emits several volatile organic compounds, including 1, 3 dimethoxy benzenes, inhibiting 62–85% growth of the tomato pathogen [191].

Similarly, benzoxazole, benzothiazole, and benzyl acetone were isolated from *B. subtilis* ZD01, strongly inhibited the growth of plant pathogen *Alterneria solani.* Moreover, the qRT-PCR results indicate that these volatile organic metabolites down-regulate the expression of fungal virulence genes i.e., *slt2* and *sod* [192]. Thus, the strain ZD01 provides a potential application as a biocontrol agent against early blight disease.

#### 6.2.5. Ketones

The biosynthesis of ketones is usually the result of fatty acid decarboxylation. Acetoin and its oxidative form butanedion are synthesized during the anaerobic fermentation of pyruvate. The two pyruvate molecules are condensed and converted to acetolactate by the acetolactate synthase enzyme. The acetolactate is further decarboxylated to form acetoin. Ketones are mainly known to inhibit the growth of plant pathogenic fungi. However, its antibacterial activity is yet not reported. Two ketones metabolites, i.e., 2-decanone, and 2-nonanone, displayed 100% growth inhibition of *F. oxysporum* [193]. In contrast, pentadecanone and 2-tetradecanone displayed bulky peaks on GCs but did not exhibit significant antifungal activity [193].

Recently, five ketones metabolites were collected from *B. velezensis* culture, and their antifungal activity was evaluated. The ketone metabolite 6-methyl 5-hepten 2-nonadecanone was revealed to inhibit 85.59% of the growth of *A. solani*, while 2-tetradecanone and 2-nonadecanone only showed mild antifungal activity [194]. The investigators further demonstrated that the antifungal activity of volatile metabolites might not be correlated with the number of carbon atoms in the metabolite [193,194]. In addition to its antimicrobial activity, ketone VOCs may also perform some other important functions. For example, acetone and butanone have been associated with plant growth and stress tolerance and induce systemic resistance [195].

#### 6.2.6. Hydrocarbon Metabolites

Hydrocarbon metabolites include alkane, alkene, and alkyne metabolites usually derived from fatty acid degradation via elongation decarboxylation or head-to-head condensation. Hydrocarbons are the most stable volatile organic metabolites and tend to remain in their original architecture over a long period of time. They may be used as a biomarker to estimate the age of primeval bacteria [196]. The *B. subtilis* group secretes various types of hydrocarbons such as nonane, tridecane, tetradecane, 2-methylpropane, and cyclohexane. Hydrocarbons like alkane, alkene, nonane, and decane are gaining particular interest due to their use as antimicrobial agents and fossil fuels. Nonane and 8-methyl heptadecane were isolated from *B. velezensis* and exhibited antifungal activity against several fungal pathogens [197]. Likewise, 1 3-butadiene inhibits the growth of phytopathogen and, additionally, negatively influences the chemotoxicity of the fungal pathogens [198].

#### 6.2.7. Organic Acids

Volatile organic acids are less abundantly produced as compared to benzenoids and ketones by *B. subtilis* group. However, several strains have been reported to emit beneficial organic acids. For instance, acetic acid and oleic acid emitted by the *B. subtilis* group are used as flavoring and preservative agents in food industries.

Acetic acid and 2-methyl propionic acid were isolated from *B. amyloliquefaciens* and were investigated for their antifungal activities. The authors find that these organic acids significantly inhibit the growth of *F. oxysporum* and *M. perniciosa* [199]. The endophytic *B. subtilis* strain DZSY21 was identified as inhibiting the growth of the maize leaf spot pathogen *Curvalaria lunata*, [154]. The growth inhibition towards *C. lunata* was observed in a time-dependent manner, i.e., the inhibition started on the 3^rd^ day and reached 36.2% on day seven. The conjoint analysis of antagonistic activity and GC-MS identified that volatile organic metabolites, including isopentyl acetate and 2-methyl butyric acid, are responsible for antifungal activity [154].

#### 6.2.8. Other Volatile Organic Metabolites

Besides the classes of volatile organic metabolites, the *B. subtilis* group also produces alkanes, alkenes, aldehydes, esters, and furans. Recently, Marco kai summarized that the *B. subtilis* group emits 15% ketones, 14% nitrogen-containing metabolites, hydrocarbons, aromatic metabolites, 11% benzenoid and alcohols, 7% organic acids, 6.5% each aldehyde and esters, and 3% sulfur-containing metabolites [12]. The alkenes (1H-indene, 1-methylene) and Furan, 2-pentyl were previously isolated from *B. subtilis* and *B. pumilus* strains and displayed significant antifungal activity against soil-born plant pathogenic fungi. However, the sclerotium and mycelial plugs grew normally in a fresh medium after treatment with the antifungal volatiles for one week, hindering the fungicidal activity of the volatiles [200]. This non-anti fungicidal effect may be related to its solubility in water, where the metabolites are adsorbed in an agar medium to perform their function. Aldehyde (nonanal) emitted by *B. subtilis* strain JA potentially inhibits the growth of the fruit and vegetable pathogen *Botrytis cinerea* [201]. Esters are recently reported to have antifungal and plant growth-promoting properties. Isopentyle acetate produced by *B. subtilis* strain DZSY21 was shown to inhibit the growth of plant pathogen *Culvularia lunata* [154]. The underlying molecular mechanism by which these volatiles impede the growth of fungi is still poorly understood. However, the antifungal activity of volatile metabolites could be related to the cell disruption phenomenon. It induces membrane permeability in fungal spores and decreases the transport of potassium ions into the cell [202]. In order to compensate for this imbalance, the proton efflux pump is activated to increase the flow of hydrogen ions into the cell and maintain the net charge on both sides of the membrane. This probably induces a rapid change in pH inside the cell, and disturbs cell physiology leads to death. Several studies highlighted the ability of volatile metabolites to interrupt pH gradients between extracellular and intracellular medium [203,204,205,206].

## 7. Miscellaneous Metabolites

There are few bioactive metabolites produced by *B. subtilis* that do not fit into any class. For instance, bacilysocin is neither synthesized via NRPS nor PKS. Instead, it is a phospholipid antibiotic that accumulates within the *B. subtilis* cell and presents a unique example of a modified phospholipid. Bacilysocin’s structure is composed of a central glycerol linked with glyceryl phosphate and an *anteiso*-fatty acid tail. The putative biosynthetic pathway for the bacilysocin initiate is from the conversion of phosphatidic acid to phosphatidylglycerol and then lysophospholipase (YtpA) convert phosphatidylglycerol to bacilysocin. The antimicrobial activity of bacilysocin is limited to Gram-positive bacteria and a few fungal strains, including *S. aureus*, *Candida pseudotropicalis* and *S. cerevisiae*. To date, the biological role, absolute biosynthetic pathway, mode of action, and the specific activity of bacilysocin is unclear.

*B. subtilis* strain 84R5 produces aminosaccharide 3,3′-neotrehalosadiamine, which exhibits marginal activity against *K. pneumoniea* and *S. aureus* [207]. The biosynthetic pathway and mode of action of aminosacharide 3,3-neotrehalosadiamine are yet not elucidated; however, a putative aminotransferase, associated with its biosynthesis has been purified and characterized thoroughly [208].

## 8. Conclusions and Future Prospective

Ubiquitous bacteria like *B. subtilis* are usually disregarded and their biosynthetic potential is underestimated; however, they produce promising bioactive metabolites that have earned much attention recent years. Herein, we review in-depth all of the bioactive metabolites produced by the *B. subtilis* group and described their potential applications in various industries. *B. subtilis* synthesize a plethora of biologically active metabolites exhibiting broad-spectrum antimicrobial, anticancer, anti-inflammatory, and antinematicidal activities. The bioactive metabolites produced by *B. subtilis* are also associated with the promotion of plant growth and induce systemic resistance in plants. This great versatility is intensifying the commercial interest in *B. subtilis* strains, considering their antimicrobial activity against plant and food-borne pathogens and their long history of safe use in food. The current review also proposed a classification system for the bioactive metabolites synthesized by *B. Subtilis* group. This classification system will assist in establishing robust practices for the characterization and the discovery of new bioactive metabolites. Undeniably, the majority of the reported biologically active metabolites are partially purified and may contain a mixture of metabolites, or the biosynthetic pathways have been identified via genome mining approaches. Therefore, the bioactivity of individual metabolites and the confirmation of particular pathways via gene knockout is required. Further, the concentrations of the partially purified metabolites are often unknown, and it is also rarely reported that the bioactive metabolites within the mixture displayed a synergistic effect. The newly isolated bioactive metabolites need proper identification, characterization, and classification in these cases.

Collectively, the biologically active metabolites produced by *B. subtilis* present a resilient foundation for developing this species to be utilized in the agronomy, food, and pharmaceutical industries. Nonetheless, the isolation and purification of these metabolites presents a primary challenge, and, also, the isolated yields and their direct applications may not be viable.

## Data Availability

No new data were created or analyzed in this study. Data sharing is not applicable to this article.

## Supplementary Materials

The following supporting information can be downloaded at: https://www.mdpi.com/article/10.3390/molecules28030927/s1, Figure S1: Showing various confirmations of surfactin and iturin cyclic lipopeptides; S2: Showing molecular structure of gageopeptide (gageostatin A, B and C) (A) and siderophore (bacilysin, chlorotetain, and bacillibactin) linear lipopeptide (B and C); S3: Chemical structure of polyketides (PKs), difficidine (A), aurantinin (B) and macrolactin (C) and Hybrid NRPs/PKs metabolites bacillaene (D) and amicoumacin A and B (E) and amicoumacin C (F); S4: Representing structure of volatile bioactive metabolites produced by *B. subtilis* group. Table S1: NRPs from *B. subtilis* group.; S2: Ribosomal peptides (RPs) from *B. subtilis* group; S3: PolyKetides (PKs) from *B. subtilis* group; S4: Hybrid (NRP/PK) metabolites from *B. subtilis* group; S5: Volatile metabolites from *B. subtilis* group. References [209,210,211,212,213,214,215,216,217,218,219,220,221,222,223,224,225,226,227,228,229,230,231,232,233,234,235,236,237,238,239,240,241,242,243,244,245,246,247,248,249,250,251,252,253,254,255,256,257,258,259,260,261,262,263,264,265] are cited in the supplementary materials.
