# Supplementary material for: Classification and Multifaceted Potential of Secondary Metabolites Produced by Bacillus subtilis Group: A Comprehensive Review
Source: Molecules. 2023 Jan 17;28(3):927. doi: 10.3390/molecules28030927 (PMC9919246; doi:10.3390/molecules28030927)
Supplement: Supplementary file 1 [file molecules-28-00927-s001.zip › molecules-2131203-supplementary.pdf]

Classification and Multifaceted potential of secondary Metabolites Produced by *Bacillus subtilis* group: A Comprehensive Review

Sajid Iqbal[1*], Farida Begum[2], Ali A. Rabaan[3,4,5,], Mohammed Aljeldah[6], Basim R. Al Shammari[6], Abdulsalam Alawfi[7], Amer Alshengeti[7, 8], Tarek Sulaiman[9], Alam Khan[10]

Affiliations:

[1] Atta-ur-Rahman School of Applied Biosciences, National University of Sciences and Technology (NUST), Islamabad, Pakistan

[2] Department of Biochemistry, Abdul Wali Khan University Mardan (AWKUM), Khyber Pakhtunkhwa, Pakistan

[3] Molecular Diagnostic Laboratory, Johns Hopkins Aramco Healthcare, Dhahran 31311, Saudi Arabia

[4] College of Medicine, Alfaisal University, Riyadh 11533, Saudi Arabia

[5] Department of Public Health and Nutrition, The University of Haripur, Haripur 22610, Pakistan

[6] Department of Clinical Laboratory Sciences, College of Applied Medical Sciences, University of Hafr Al Batin, Hafr Al Batin 39831, Saudi Arabia

[7] Department of Pediatrics, College of Medicine, Taibah University, Al-Madinah 41491, Saudi Arabia

[8] Department of Infection prevention and control, Prince Mohammad Bin Abdulaziz Hospital, National Guard Health Affairs, Al-Madinah 41491, Saudi Arabia

[9] Infectious Diseases Section, Medical Specialties Department, King Fahad Medical City, Riyadh 12231, Saudi Arabia.

[10] Department of Life Sciences, Abasyn University Islamabad campus, Islamabad, Pakistan

[*]Corresponding: Sajid Iqbal

Email; siqbal.phdabs12asab@asab.nust.edu.pk; sajidiqbalmb44@yahoo.com

Phone number: +923139591716

Table S1: NRPs from *B. subtilis* group.

NRPs class	Sub-class	Metabolites	Antimicrobial activity				References
			Antibacterial		Antifungal	Other	
			Gram +ive	Gram -ive			
Cyclic lipopeptide	Fengycin	Agrastatin A	-	-	+	-	[1]
Cyclic lipopeptide	Fengycin	Fengycin	-	-	+	-	[2,3,4]
Cyclic lipopeptide	Fengycin	Fengycin A	-	-	+	-	[5]
Cyclic lipopeptide	Fengycin	Fengycin B	-	-	+	-	[6]
Cyclic lipopeptide	Fengycin	Fengycin C, D, S	-	-	+	-	[5]
Cyclic lipopeptide	Fengycin	Plipastatin	-	-	+	-	[7]
Cyclic lipopeptide	Iturin	Bacillomycin	-	-	+	-	[8,9]
Cyclic lipopeptide	Iturin	Bacillomycin D	-	-	+	-	[10]
Cyclic lipopeptide	Iturin	Bacillomycin F	-	-	+	-	[11]
Cyclic lipopeptide	Iturin	Bacillomycin L	-	-	+	-	[12]
Cyclic lipopeptide	Iturin	Bacillomycin Lc	-	-	+	-	[13]
Cyclic lipopeptide	Iturin	Bacillomycin R	+	-	+	-	[13]
Cyclic lipopeptide	Iturin	Eumycin	-	-	+	-	[13]
Cyclic lipopeptide	Iturin	Iturin	-	-	+	-	[2,14]
Cyclic lipopeptide	Iturin	Iturin A	-	-	+	-	[6,12]
Cyclic lipopeptide	Iturin	Iturin C	-	-	+	-	[14]
Cyclic lipopeptide	Iturin	Iturin D, E	-	-	+	-	[13]
Cyclic lipopeptide	Iturin	Mycosubtilin	+	-	+	-	[12,15]
Cyclic lipopeptide	Iturin	Subtulene A	+	+	+	-	[16]
Cyclic lipopeptide	Surfactin	Bamylocin A	-	-	+	-	[11,17,18]
Cyclic	Surfactin	Lichenysin	+	+	+	-	[19]

lipopeptide							
Cyclic lipopeptide	Surfactin	Lichenysin A	+	+	-	-	[20]
Cyclic lipopeptide	Surfactin	Locillomycin	+	+	-	Antiviral	[21]
Cyclic lipopeptide	Surfactin	Pumilacidin A-G	+	+	-	Antiviral	[22]
Cyclic lipopeptide	Surfactin	Surfactin	+	+	+	-	[2,23,24]
Cyclic lipopeptide	Surfactin	fengycin	-	-	+	-	[22,25,26,27]
Cyclic lipopeptide	Surfactin	Surfactin like peptide	+	+	+	-	[28,29]
Cyclic lipopeptide	-	Gageopeptine	-	-	+	-	[30]
Cyclic lipopeptide	-	Bacilotetrins A and B	+	-	-	-	[30]
Cyclic lipopeptide	Nonapeptide	Locillomycins	+	+	-	Antiviral	[21]
Linear lipopeptide	Gageopeptid e	Gageotetrine A-C	-	-	+	-	[30]
Linear lipopeptide	-	Gageostatins	-	-	+	-	[30]
Linear lipopeptide	Siderophore	Bacillibactin	-	-	+	+	[31]
Other NRPs	Dipeptide	Bacilysin	+		+	-	[32]
Other NRPs	cyclohexeno ne	Chlorotetain	-	-	+	-	[33]
Other NRPs	Polypeptide	Bacitracin A, F	-	+	-	-	[34]
Other NRPs	Polypeptide	Mycobacillin	-	-	+	-	[35]
Other NRPs	Dipeptide	Rhizocticin A	-	-	+	-	[36]

Table S2: Ribosomal peptides (RPs) from *B. subtilis* group.

RPs Class	Sub-class	Metabolite	Antimicrobial activity			References
			Antibacterial activity		Antifungal activity	
			Gram +ive	Gram -ive		
Modified peptides	Lanthibiotics	Entianin	+	-	-	[37,38,39,40]
Modified peptides	Lanthibiotics	Ericin A	+	-	-	[41]
Modified peptides	Lanthibiotics	Ericin S	+	-	-	[41,42]
Modified peptides	Lanthibiotics	Subtilin	+	-	-	[41,43]
Modified peptides	Lanthibiotics	Mersacidin	+	-	-	[44]
Modified peptides	Lanthibiotics	Sublancin 168	+	-	-	[45]

Modified peptides	Lanthibiotics	Lichenicidin	+	-	-	[46]
Modified peptides	Lanthibiotics	Subtilosin A	+	+	-	[47,48,49]
Modified peptides	Lanthibiotics	Subtilomycin	+	+	+	[50,51]
Others	Bacteriocin like peptides	Lichenin	+	-	-	[48]
Non-modified peptides	Large antimicrobial peptides	Bac 14B	+	+	+	[49]
Non-modified peptides	Large antimicrobial peptides	Baciamin	-	-	+	[50]
Modified peptides	Cyclized peptides	Amylocyclicin	+	-	-	[52]
Modified peptides	LAP	Sonorensin	+	+	-	[53]
Modified peptides	LAP	Plantazolicin	+	-	-	[52]
Non-modified peptide	Large antimicrobial peptide	CAMT2	+	+	-	[51]
Non-modified peptide	Large antimicrobial peptide	Bacisubin	-	-	+	[54]
Modified peptides	Lanthibiotics	Amylolysin	+	+	+	[55]

Table S3: PolyKetides (PKs) from *B. Subtilis*

**PKs Class**	**Sub-class**	**Metabolite**	**Antibacterial activity**		**Antifungal activity**	**Other**	**References**
			**Gram +ive**	**Gram -ive**			
Polyene	Macrolactin	7-O-6′(2″-acetylphenyl)-5′-hydroxyhexanoatemacrolactin A	**-**	+	-	-	[56]
Polyene	Macrolactin	7-O-2′E-butenoyl-macrolactin A	**-**	**-**	+	-	[5]
Polyene	Macrolactin	Lactones with 3 diene moieties in the carbon	+	-	-	Antiviral	[8]

		backbone					
Polyene	Macrolactin	Gageomacrolactins	+	-	-	-	[30]
Polyene	Macrolactin	7-O-methyl-5′-hydroxy-3′-heptenoate-macrolactin A	+	+	-	-	[56]
Polyene	Macrolactin	7-*O*-malonyl-macrolactin A	+	+	+	-	[8]
Polyene	Macrolactin	7-*O*-succinyl-macrolactin F	+	+	-	-	[57]
Polyene	Macrolactin	7-*O*-succinyl-macrolactin A	+	+	+	-	[57]
Polyene	Macrolactin	Macrolactin A	+	+	+	-	[58]
Polyene	Macrolactin	7-*O*-2′*E*-butenoyl macrolactin A	-	-	+	-	[5]
Polyene	Macrolactin	Macrolactin B and C	+	-	-	-	[59]
Polyene	Macrolactin	Macrolactin D	+	+	+	-	[21]
Polyene	Macrolactin	Macrolactin F-M	+	-	-	-	[57]
Polyene	Macrolactin	Macrolactin N	+	+	-	-	[31]
Polyene	Macrolactin	Macrolactin Q	+	+	-	-	[60]
Polyene	Macrolactin	Macrolactin S	+	+	+	-	[61]
Polyene	Macrolactin	Macrolactin T	+	-	+	-	[5]
Polyene	Macrolactin	Macrolactin W	+	-	-	-	[60]
Polyene	-	Aurantinin B-D	+	-	-	-	[62]
Polyene	-	2-(8-butyl-3-ethyl-3,4,4a,5,6,8a-hexahydro-2H-chromen-6-yl)-ethyl benzoate	-	+	-	-	[56]
Polyene	-	methoxycarbonyl)-4-(5-(2-ethylbutyl)-5,6-dihydro-3-methyl-2H-pyran-2-yl)-butyl benzoate	-	+	-	-	[56]
Polyene	Difficidin	Macrocyclic	+	+	-	-	[63]

		polyene lactone phosphate ester					
Polyene	Difficidin	Difficidin	+	+	-	-	[63]
Polyene	Difficidin	Oxydifficidin	+	+	-	-	[64]

Table S4: Hybrid (NRP/PK) metabolites from *B. Subtilis.*

Class	Sub-class	Metabolite	Antibacterial activity		Antifungal activity	Other activity	References
			Gram +ive	Gram -ive			
Hybrid.	Bacillaene.	Bacillaene A.	+.	+.	+.	-.	[65]..
Hybrid.	Isocoumarins. .	N-acetylated amicoumacin B.	-.	+.	-.	-.	[59] ..
Hybrid.	Isocoumarins.	Amicoumacin A.	+.	-.	-.	Anticancer, Antiulcer and Anti-inflammatory.	[5]..
Hybrid.	Isocoumarins.	Amicoumacin A B, C.	+.	-.	-.	Anti-inflammatory.	[59]..
Hybrid.	Isocoumarins.	AI-77s.	+.	-.	-.	Gastro protective .	[66] ..
Hybrid.	Isocoumarins.	Baciphelacin.	+.	+.	-..		[67] ..
Hybrid.	Isocoumarins.	Bacilosarcins A and B.	+.	+.	-.	herbicidal.	[68]..
Hybrid.	Isocoumarins.	Damxungmacin A and B.	+..		-.	Anticancer.	[69]..
Hybrid.	Isocoumarins.	PM-94128.	-.	-.	-.	Anticancer.	[70]..
Hybrid.	Isocoumarins.	Hetiamacin A (PJS).	+.	-.	-.	-.	[54]..
Hybrid.	Isocoumarins.	Hetiamacin B.	+.	-.	-.	-.	[54]..
Hybrid.	Isocoumarins.	Lipoamicoumacins A-D.	+.	+.	-.	Anticancer.	[5]..

Table S5: Volatile metabolites from *B. subtilis* group.

Class	Sub-class	Metabolite	Antifungal activity	Antibacterial activity	References
Organic metabolite	Acids	Butanoic acid, 3-methyl	+	-	[37]
Organic metabolite	Acids	Gentisic acid	+	-	[38]
Organic metabolite	Acids	n-Hexanoic acid	-	+	[39]
Organic metabolite	Acids	n-Hexadecanoic acid	+	-	[38]

Organic metabolite	Acids	Octadecanoic acid, Propanoic acid, 4-hexen-1-yl ester	+	-	[54]
Organic metabolite	Acids	Oleic acid	-	+	[39]
Organic metabolite	Acids	Propanoic acid, 2-methyl	+	-	[37]
Organic metabolite	Acid	Isovaleric acid	+	-	[71]
Organic metabolites	Acid	2-methylbutyric acid	+	-	[32]
Organic metabolites	Alcohols	Ethanol	+	-	[72]
Organic metabolite	Alcohols	3,4-dimethyl-5-hexen-3-ol, Heptanol	+	-	[54]
Organic metabolite	Alcohal	1-decanol	+	-	[73]
Organic metabolite	Alcohols	2-Undecanol	+	-	[22]
Organic metabolite	Alcohols	1-Butanol	+	-	[37]
Organic metabolite	Alcohols	1-Butanol, 3-methyl-	+	-	[37,54]
Organic metabolite	Alcohols	Hexanol,2-ethyl	+	-	[63]
Organic metabolite	Alcohal	Isoamyl alcohol/3-methyl-1-butanol	+	-	[71]
Organic metabolite	Alcohal	1-dodecanol	+	-	[73]
Organic metabolite	Acohal	2-ethylhexanol	+	-	[74]
Organic metabolite	Aldehyde	2,4-Heptadienal, (E,E)-, 2-Decenal, (E)-, 2-Heptenal, (Z)-, 2-Nonenal, (E)-, 2-Octenal, (E)-, 2-Undecenal, 2,4 Decadienal, Octanal	+	-	[54]
Organic metabolite	Aldehyde	Decanal	+	-	[58]
Organic metabolite	Aldehyde	Nonanal	+	-	[39]
Organic metabolites	Hydrocarbon	5-methyl-2-heptanone	+	-	[72]
Organic metabolite	Hydrocarbon	Eicosane, 10-methyl-	+	-	[63]
Organic metabolite	Hydrocarbon	Heneicosane	+	-	[38]
Organic metabolite	Hydrocarbon	Heptadecane	-	+	[39]
Organic metabolite	Hydrocarbon	Heptadecane, 2,6,10,15-tetramethyl-	+	-	[63]
Organic metabolite	Hydrocarbon	Heptane, 2-methyl-7-oxabicyclo[2.2.1]	+	-	[54]
Organic metabolite	Hydrocarbon	Hexadecane, 2,6,11,15-tetramethyl-	+	-	[63]
Organic metabolite	Hydrocarbon	Nonadecane, 9-methyl-	+	-	[63]
Organic metabolite	Hydrocarbon	Nonadecane,10-methyl-	+	-	[63]
Organic metabolite	Hydrocarbon	Pentadecane	+	-	[75]
Organic metabolite	Hydrocarbon	Pentadecane, 8-hexyl-	+	-	[63]

Organic metabolite	Hydrocarbon	Tetradecane	+	-	[75]
Organic metabolite	Hydrocarbon	Tetradecane, 2,6,10-trimethyl-	+	-	[63]
Organic metabolite	Hydrocarbon	Undecane,1,2-methyl	+	-	[75]
Organic metabolite	Hydrocarbon	1H-indene, 1-methylene-	+	-	[54]
Organic metabolite	Hydrocarbon	1,3-butadiene	+	+	[76]
Organic metabolite	Hydrocarbon	8-methyl-1-decene	+	-	[54]
Organic metabolite	Htdrocarbon	m-tolunitrile	+	-	[74]
Organic metabolite	Hydrocarbon	1,3- pentadiene	+	-	[77]
Organic metabolite	Hydrocarbon	6-methyl-2-heptanone	+	-	[74]
Organic metabolite	Benzenoids	β-Benzeneethanamine	+	-	[38]
Organic metabolite	Benzenoids	1,2-Benzisothiazol-3(2H)-one	-	+	[76]
Organic metabolite	Benzenoids	2,4-bis(2-Methylpropyl)-phenol	+	-	[63]
Organic metabolite	Benzenoids	2-Phenylethanol	-	+	[39]
Organic metabolite	Benzenoids	4-Hydroxybenzaldehyde	+	+	[76]
Organic metabolite	Benzenoids	Benzene, 1,2,4,5-tetramethyl	+	-	[63]
Organic metabolite	Benzenoids	Benzene, 1,2,4-trimethyl	+	-	[58]
Organic metabolite	Benzenoids	Benzene, 1-methyl-4-(1-methylethyl)-	+	-	[58]
Organic metabolite	Benzenoids	Benzene, 2-propenyl	+	-	[75]
Organic metabolite	Benzenoids	Benzene,1,4-dichloro	+	-	[75]
Organic metabolite	Benzenoids	Benzothiazole	+	-	[74]
Organic metabolite	Benzenoids	Butylated hydroxytoluene	+	-	[38]
Organic metabolite	Benzenoids	Ethylbenzene	+	-	[58]
Organic metabolite	Benzenoids	Phenol, 2,4-bis(1,1-dimethylethyl)	+	-	[7]
Organic metabolite	Benzenoids	Phenol, 4,4′-(1-methylethylidene) bis-	-	+	[39]
Organic metabolite	Benzenoids	Phenol, 4-chloro-3-methyl	+	-	[7]
Organic metabolite	Benzenoids	Phenol,2,3,6-trimethyl-	+	-	[58]
Organic metabolite	Benzenoids	P-xylene	+	-	[58]
Organic metabolite	Benzenoids	Styrene	+	-	[22]
Organic metabolite	Benzenoids	Toluene	+	-	[39]
Organic metabolite	Benzenoids	Phenol	+	-	[73]
Organic metabolite	Benzenoids	2,4-di-*tert*-butylphenol	+	+	[7]
Organic metabolite	Benzenoid	Benzylacetone	+	-	[74]
Organic metabolite	Esters	Ethyl acetate	+	-	[37]
Organic metabolite	Ester	Butyl formate	+	-	[71,78]

Organic metabolite	Ester	Isopentyl acetate	+	-	[79]
Organic metabolite	Furans	2-pentyl-furan	+	-	[54]
Organic metabolite	Ketone	2-nonanone	+	-	[74,80]
Organic metabolite	Ketone	Acetophenone	+	-	[74]
Organic metabolites	Ketone	Methyl isobutyl ketone	+	-	[72]
Organic metabolite	Ketone	Acetoin	+	-	[77]
Organic metabolite	Ketone	2-heptanone	+	-	[79,80]
Organic metabolite	Ketone	Butan-2-one	+	-	[37]
Organic metabolite	Ketone	Butanone, 3-hydroxy-2-	+	-	[37]
Organic metabolite	Ketone	Dodecan-2-one	+	-	[38]
Organic metabolite	Ketone	Ethanone, 1-(4-methylphenyl)	+	+	[39]
Organic metabolite	Ketone	Pentanone, 2,2,4-trimethyl-3-	-	+	[5]
Organic metabolite	Ketone	Propan-2-one	+	-	[37]
Organic metabolite	Ketone	Tridecan-2-one	+	-	[39]
Organic metabolite	Ketone	5-methyl-2-hexanone	+	-	[74]
Organic metabolite	Nitrogen-containing	1-ethyl-1H-imidazole	+	-	[54]
Organic metabolite	Nitrogen-containing	Ammonium acetate	+	-	[63]
Organic metabolite	Nitrogen-containing	2,5-dimethyl Pyrazine	+	-	[78]
Organic metabolite	Nitrogen-containing	2-ethyl-3,5-dimethyl Pyrazine	+	-	[37]
Organic metabolite	Nitrogen-containing	Tetramethyl-Pyrazine	+	-	[63]
Organic metabolite	Nitrogen-containing	2,3,5,6-tetramethyl Pyrazine	+	-	[37]
Organic metabolite	Nitrogen-containing	S-(−)-2-methylbutylamine	+	-	[72]
Organic metabolite	Nitrogen-containing	2,3-dimethyl pyrazine	+	-	[71]
Organic metabolite	Sulphur-containing	Carbon disulphide	-	+	[37]
Organic metabolite	Sulphur-containing	Dimethyl trisulfide	+	-	[81]
Organic metabolite	Hydrocarbon	Nonane	+	-	[71]
Organic metabolite	Aldehyde	Benzaldehyde	+	-	[73]
Organic metabolite	Ketones	Diacetyl/Butane-2,3-dione	+	-	[71]
Organic metabolite	Sulphur-containing	Thiophene	+	-	[77]

Inorganic metabolite	-	Hydrogen sulfide	+	-	[32]
Inorganic metabolites	-	Hydrogen cyanide	+	-	[73,81]

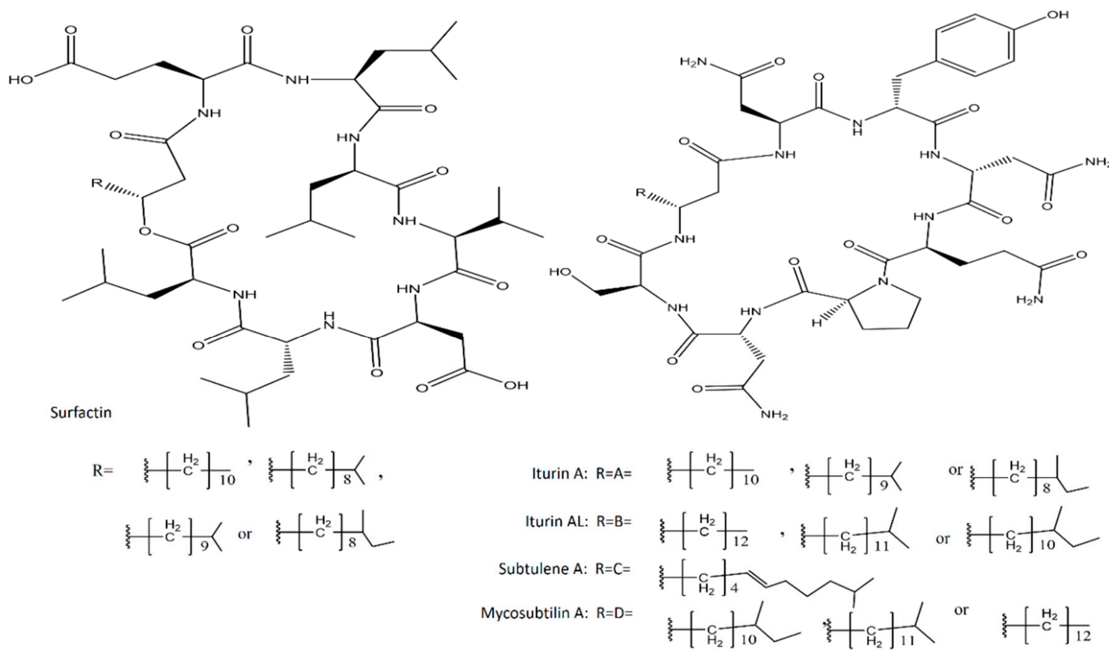

Figure S1. Showing various confirmations of surfactin and iturin cyclic lipopeptides.

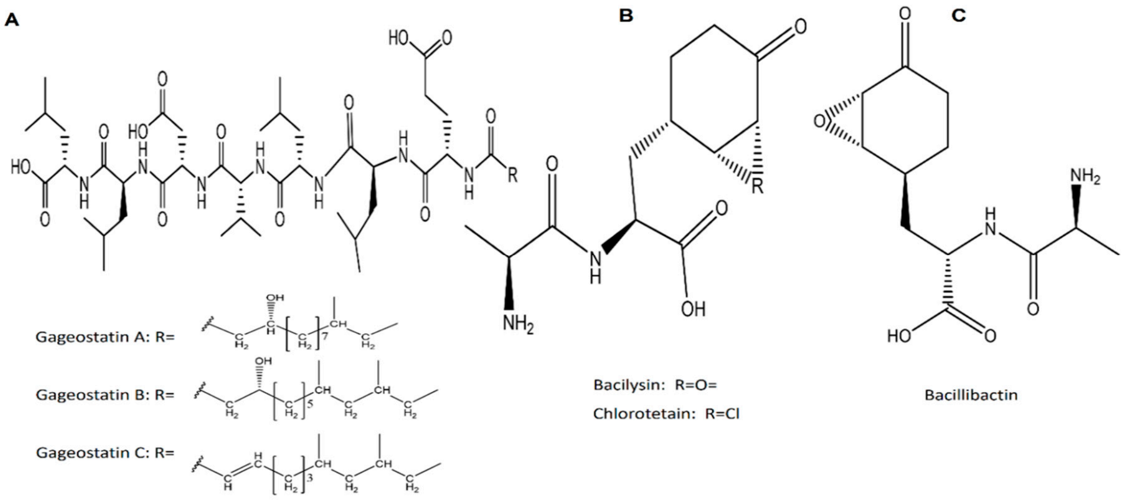

Figure S2. Showing molecular structure of gageopeptide (gageostatin A, B and C) **(A)** and siderophore (bacilysin, chlorotetain, and bacillibactin) linear lipopeptide **(B and C)**.

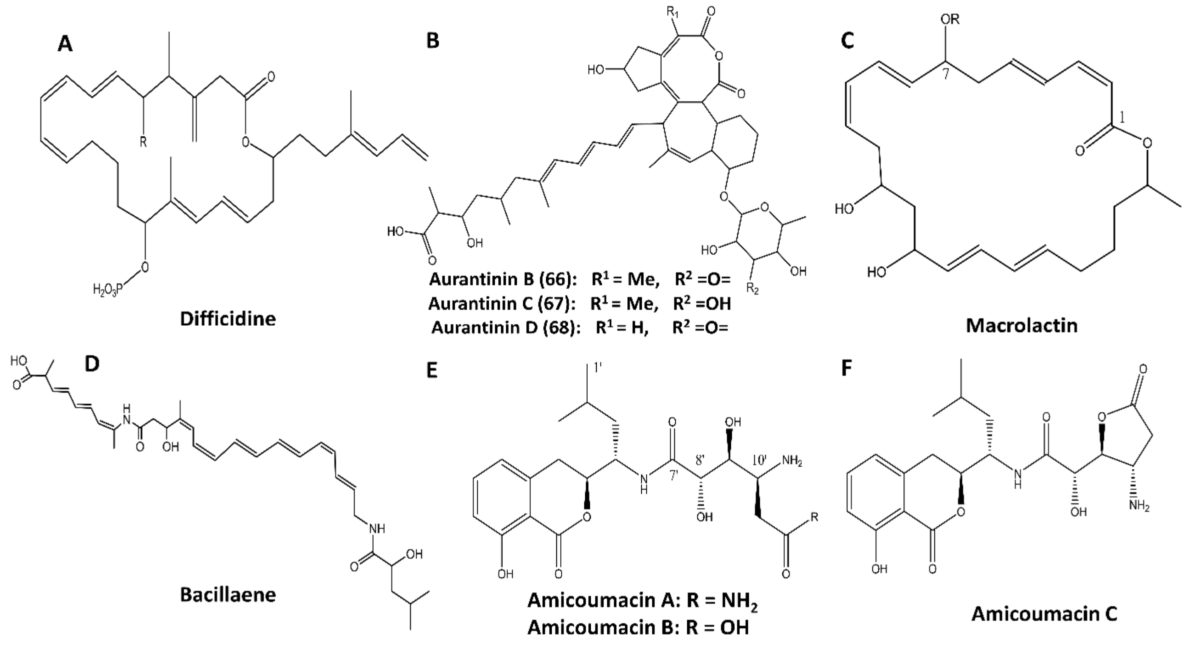

Figure S3. Chemical structure of polyketides (PKs), difficidine (A), aurantinin (B) and macrolactin (C) and Hybrid NRPs/PKs metabolites bacillaene (D) and amicoumacin A and B (E) and amicoumacin C (F).

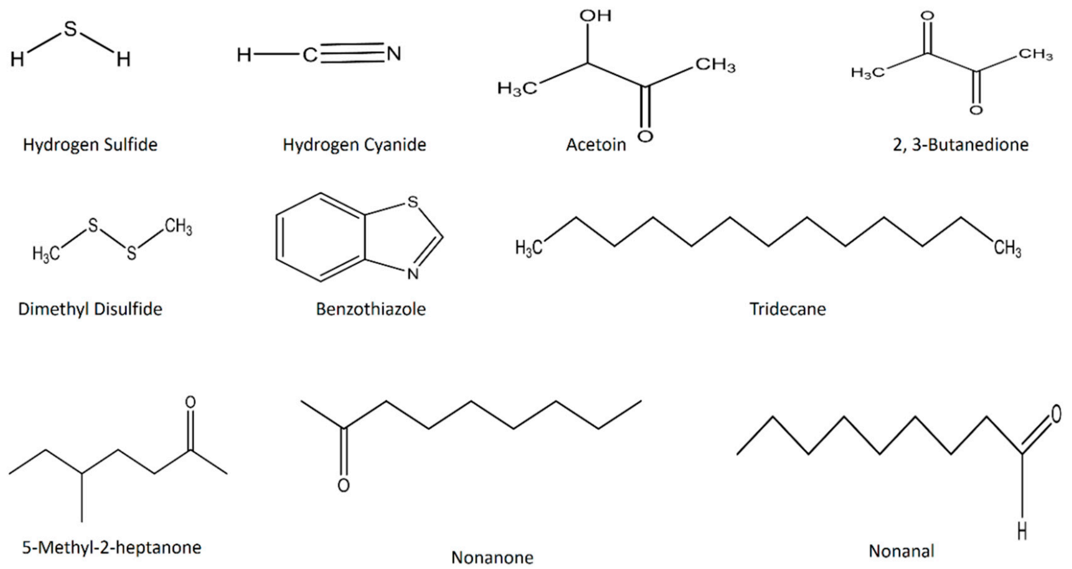

Figure S4. Representing structure of volatile bioactive metabolites produced by *B. subtilis* group.

References

1. Zhao, P.; Quan, C.; Wang, Y.; Wang, J.; Fan, S. *Bacillus amyloliquefaciens* Q-426 as a potential biocontrol agent against *Fusarium oxysporum* f. sp. *spinaciae*. *Journal of Basic Microbiology* **2014**, *54*, 448–456, doi:https://www.doi.org/10.1002/jobm.201200414.
2. Cawoy, H.; Debois, D.; Franzil, L.; De Pauw, E.; Thonart, P.; Ongena, M. Lipopeptides as main ingredients for inhibition of fungal phytopathogens by *Bacillus subtilis/amyloliquefaciens*. *Microbial Biotechnology* **2015**, *8*, 281–295, doi:https://www.doi.org/10.1111/1751-7915.12238.
3. Zhao, P.; Quan, C.; Wang, Y.; Wang, J.; Fan, S.J.J.o.b.m. *Bacillus amyloliquefaciens* Q-426 as a potential biocontrol agent against Fusarium oxysporum f. sp. spinaciae. **2014**, *54*, 448–456.
4. Wise, C.; Falardeau, J.; Hagberg, I.; Avis, T.J. Cellular Lipid Composition Affects Sensitivity of Plant Pathogens to Fengycin, an Antifungal Compound Produced by *Bacillus subtilis* Strain CU12. *Phytopathology* **2014**, *104*, 1036–1041, doi:https://www.doi.org/10.1094/PHYTO-12-13-0336-R.
5. Li, L.; Ma, M.; Huang, R.; Qu, Q.; Li, G.; Zhou, J.; Zhang, K.; Lu, K.; Niu, X.; Luo, J. Induction of Chlamydospore Formation in *Fusarium* by Cyclic Lipopeptide Antibiotics from *Bacillus subtilis* C2. *Journal of Chemical Ecology* **2012**, *38*, 966–974, doi:https://www.doi.org/10.1007/s10886-012-0171-1.
6. Malfanova, N.; Franzil, L.; Lugtenberg, B.; Chebotar, V.; Ongena, M. Cyclic lipopeptide profile of the plant-beneficial endophytic bacterium *Bacillus subtilis* HC8. *Archives of Microbiology* **2012**, *194*, 893–899, doi:https://www.doi.org/10.1007/s00203-012-0823-0.
7. Gao, L.; Han, J.; Liu, H.; Qu, X.; Lu, Z.; Bie, X. Plipastatin and surfactin coproduction by *Bacillus subtilis* pB2-L and their effects on microorganisms. *Antonie van Leeuwenhoek* **2017**, *110*, 1007–1018, doi:https://www.doi.org/10.1007/s10482-017-0874-y.
8. Romero, D.; de Vicente, A.; Rakotoaly, R.H.; Dufour, S.E.; Veening, J.-W.; Arrebola, E.; Cazorla, F.M.; Kuipers, O.P.; Paquot, M.; Pérez-García, A. The Iturin and Fengycin Families of Lipopeptides Are Key Factors in Antagonism of *Bacillus subtilis* Toward *Podosphaera fusca*. *Molecular Plant-Microbe Interactions* **2007**, *20*, 430–440, doi:https://www.doi.org/10.1094/MPMI-20-4-0430.
9. Raubitschek, F.; Dostrovsky, A. An Antibiotic Active against Dermatophytes, Derived from *Bacillus Subtilis*. *Dermatology* **1950**, *100*, 45–49, doi:https://www.doi.org/10.1159/000257151.
10. Moyne, A.-L.; Shelby, R.; Cleveland, T.E.; Tuzun, S. Bacillomycin D: An iturin with antifungal activity against *Aspergillus flavus*. *Journal of Applied Microbiology* **2001**, *90*, 622–629, doi:https://www.doi.org/10.1046/j.1365-2672.2001.01290.x.
11. Lee, S.-C.; Kim, S.-H.; Park, I.-H.; Chung, S.-Y.; Choi, Y.-L. Isolation and structural analysis of bamylocin A, novel lipopeptide from *Bacillus amyloliquefaciens* LP03 having

antagonistic and crude oil-emulsifying activity. *Archives of Microbiology* **2007**, *188*, 307–312, doi:https://www.doi.org/10.1007/s00203-007-0250-9.
12. Thimon, L.; Peyoux, F.; Maget-Dana, R.; Michel, G. Surface-active properties of antifungal lipopeptides produced by *Bacillus subtilis*. *Journal of the American Oil Chemists Society* **1992**, *69*, 92–93, doi:https://www.doi.org/10.1007/bf02635884.
13. Besson, F.; PEYPOUX, F.; MICHEL, G.; DELCAMBE, L. Characterization of iturin A in antibiotics from various strains of *Bacillus subtilis*. *The Journal of antibiotics* **1976**, *29*, 1043–1049, doi:https://www.doi.org/10.7164/antibiotics.29.1043.
14. Dunlap, C.A.; Schisler, D.A.; Price, N.P.; Vaughn, S.F. Cyclic lipopeptide profile of three *Bacillus subtilis* strains; antagonists of *Fusarium* head blight. *The Journal of Microbiology* **2011**, *49*, 603, doi:https://www.doi.org/10.1007/s12275-011-1044-y.
15. Leclère, V.; Béchet, M.; Adam, A.; Guez, J.-S.; Wathelet, B.; Ongena, M.; Thonart, P.; Gancel, F.; Chollet-Imbert, M.; Jacques, P. Mycosubtilin Overproduction by *Bacillus subtilis* BBG100 Enhances the Organism's Antagonistic and Biocontrol Activities. *Applied and Environmental Microbiology* **2005**, *71*, 4577–4584, doi:https://www.doi.org/10.1128/aem.71.8.4577-4584.2005.
16. Thasana, N.; Prapagdee, B.; Rangkadilok, N.; Sallabhan, R.; Aye, S.L.; Ruchirawat, S.; Loprasert, S. *Bacillus subtilis* SSE4 produces subtulene A, a new lipopeptide antibiotic possessing an unusual C15 unsaturated β-amino acid. *FEBS Letters* **2010**, *584*, 3209–3214, doi:https://www.doi.org/10.1016/j.febslet.2010.06.005.
17. Lee, D.W.; Kim, B.S. Antimicrobial Cyclic Peptides for Plant Disease Control. *The Plant Pathology Journal* **2015**, *31*, 1–11, doi:https://www.doi.org/10.5423/PPJ.RW.08.2014.0074.
18. Hsieh, F.-C.; Lin, T.-C.; Meng, M.; Kao, S.-S. Comparing Methods for Identifying *Bacillus* Strains Capable of Producing the Antifungal Lipopeptide Iturin A. *Current Microbiology* **2008**, *56*, 1–5, doi:https://www.doi.org/10.1007/s00284-007-9003-x.
19. Jenny, K.; Käppeli, O.; Fiechter, A. Biosurfactants from *Bacillus licheniformis*: Structural analysis and characterization. *Applied Microbiology and Biotechnology* **1991**, *36*, 5–13, doi:https://www.doi.org/10.1007/bf00164690.
20. Yakimov, M.M.; Timmis, K.N.; Wray, V.; Fredrickson, H.L. Characterization of a new lipopeptide surfactant produced by thermotolerant and halotolerant subsurface *Bacillus licheniformis* BAS50. *Applied and Environmental Microbiology* **1995**, *61*, 1706–1713, doi: .
21. Luo, C.; Liu, X.; Zhou, X.; Guo, J.; Truong, J.; Wang, X.; Zhou, H.; Li, X.; Chen, Z. Unusual biosynthesis and structure of locillomycins from *Bacillus subtilis* 916. *Applied and Environmental Microbiology* **2015**, doi:https://www.doi.org/10.1128/aem.01639-15.
22. Qi, G.; Zhu, F.; Du, P.; Yang, X.; Qiu, D.; Yu, Z.; Chen, J.; Zhao, X. Lipopeptide induces apoptosis in fungal cells by a mitochondria-dependent pathway. *Peptides* **2010**, *31*, 1978–1986, doi:https://www.doi.org/10.1016/j.peptides.2010.08.003.
23. Naruse, N.; Tenmyo, O.; Kobaru, S.; Kamei, H.; Miyaki, T.; Konishi, M.; Oki, T. Pumilacidin, a complex of new antiviral antibiotics. *The Journal of antibiotics* **1990**, *43*, 267–280, doi:https://www.doi.org/10.7164/antibiotics.43.267.
24. Bais, H.P.; Fall, R.; Vivanco, J.M. Biocontrol of *Bacillus subtilis* against Infection of *Arabidopsis* Roots by *Pseudomonas syringae* Is Facilitated by Biofilm Formation and Surfactin Production. *Plant Physiology* **2004**, *134*, 307–319, doi:https://www.doi.org/10.1104/pp.103.028712.

25. Preecha, C.; Sadowsky, M.J.; Prathuangwong, S. Lipopeptide surfactin produced by *Bacillus amyloliquefaciens* KPS46 is required for biocontrol efficacy against *Xanthomonas axonopodis* pv. *Glycines*. *Kasetsart Journal, Natural Sciences* **2010**, *Vol.44 No.1*, pp.84-99 doi: .
26. Dimkić, I.; Živković, S.; Berić, T.; Ivanović, Ž.; Gavrilović, V.; Stanković, S.; Fira, D. Characterization and evaluation of two *Bacillus* strains, SS-12.6 and SS-13.1, as potential agents for the control of phytopathogenic bacteria and fungi. *Biological Control* **2013**, *65*, 312–321, doi:https://www.doi.org/10.1016/j.biocontrol.2013.03.012.
27. Romano, A.; Vitullo, D.; Senatore, M.; Lima, G.; Lanzotti, V. Antifungal Cyclic Lipopeptides from *Bacillus amyloliquefaciens* Strain BO5A. *Journal of Natural Products* **2013**, *76*, 2019–2025, doi:https://www.doi.org/10.1021/np400119n.
28. Sabaté, D.C.; Audisio, M.C. Inhibitory activity of surfactin, produced by different *Bacillus subtilis* subsp. subtilis strains, against *Listeria monocytogenes* sensitive and bacteriocin-resistant strains. *Microbiological Research* **2013**, *168*, 125–129, doi:https://www.doi.org/10.1016/j.micres.2012.11.004.
29. Sharma, S.; Pandey, L.M. Production of biosurfactant by *Bacillus subtilis* RSL-2 isolated from sludge and biosurfactant mediated degradation of oil. *Bioresource Technology* **2020**, *307*, 123261.
30. Tareq, F.S.; Lee, M.A.; Lee, H.-S.; Lee, J.-S.; Lee, Y.-J.; Shin, H.J. Gageostatins A–C, antimicrobial linear lipopeptides from a marine *Bacillus subtilis*. *Marine Drugs* **2014**, *12*, 871–885.
31. Yu, X.; Ai, C.; Xin, L.; Zhou, G. The siderophore-producing bacterium, *Bacillus subtilis* CAS15, has a biocontrol effect on *Fusarium* wilt and promotes the growth of pepper. *European Journal of Soil Biology* **2011**, *47*, 138–145, doi:https://www.doi.org/10.1016/j.ejsobi.2010.11.001.
32. Wu, L.; Wu, H.; Chen, L.; Xie, S.; Zang, H.; Borriss, R.; Gao, X. Bacilysin from *Bacillus amyloliquefaciens* FZB42 has Specific Bactericidal Activity against Harmful Algal Bloom Species. *Applied and Environmental Microbiology* **2014**, doi:https://www.doi.org/10.1128/aem.02605-14.
33. Zuber, P.; Nakano, M.M.; Marahiel, M.A. Peptide Antibiotics. In *Bacillus subtilis and Other Gram-Positive Bacteria*; American Society of Microbiology: 1993.
34. Hussein, A.; AL-Janabi, S. Identification of bacitracin produced by local isolate of *Bacillus licheniformis*. *African journal of Biotechnology* **2006**, *5*.
35. Majumdar, S.K.; Bose, S.K. Mycobacillin, a New Antifungal Antibiotic produced by *B. subtilis*. *Nature* **1958**, *181*, 134, doi:https://www.doi.org/10.1038/181134a0.
36. Kugler, M.; Loeffler, W.; Rapp, C.; Kern, A.; Jung, G. Rhizocticin A, an antifungal phosphono-oligopeptide of *Bacillus subtilis* ATCC 6633: Biological properties. *Archives of Microbiology* **1990**, *153*, 276–281, doi:https://www.doi.org/10.1007/bf00249082.
37. Chaves-López, C.; Serio, A.; Gianotti, A.; Sacchetti, G.; Ndagijimana, M.; Ciccarone, C.; Stellarini, A.; Corsetti, A.; Paparella, A. Diversity of food-borne *Bacillus* volatile compounds and influence on fungal growth. *Journal of Applied Microbiology* **2015**, *119*, 487–499, doi:https://www.doi.org/10.1111/jam.12847.
38. Zheng, M.; Shi, J.; Shi, J.; Wang, Q.; Li, Y. Antimicrobial effects of volatiles produced by two antagonistic *Bacillus* strains on the anthracnose pathogen in postharvest mangos. *Biological Control* **2013**, *65*, 200–206, doi:https://www.doi.org/10.1016/j.biocontrol.2013.02.004.

39. Raza, W.; Ling, N.; Yang, L.; Huang, Q.; Shen, Q. Response of tomato wilt pathogen *Ralstonia solanacearum* to the volatile organic compounds produced by a biocontrol strain *Bacillus amyloliquefaciens* SQR-9. *Scientific Reports* **2016**, *6*, 24856, doi:https://www.doi.org/10.1038/srep24856.
40. Fuchs, S.W.; Jaskolla, T.W.; Bochmann, S.; Kötter, P.; Wichelhaus, T.; Karas, M.; Stein, T.; Entian, K.-D. Entianin, a Novel Subtilin-Like Lantibiotic from *Bacillus subtilis* DSM 15029[T] with High Antimicrobial Activity. *Applied and Environmental Microbiology* **2011**, doi:https://www.doi.org/10.1128/aem.01962-10.
41. Stein, T.; Borchert, S.; Conrad, B.; Feesche, J.; Hofemeister, B.; Hofemeister, J.; Entian, K.-D. Two different lantibiotic-like peptides originate from the ericin gene cluster of *Bacillus subtilis* A1/3. *J Bacteriol* **2002**, *184*, 1703–1711, doi:https://www.doi.org/10.1128/JB.184.6.1703-1711.2002.
42. Agrios, G.N. 1 - INTRODUCTION TO PLANT PATHOLOGY. In *Plant Pathology (Third Edition)*, Agrios, G.N., Ed.; Academic Press: 1988; pp. 3-39.
43. Heinzmann, S.; Entian, K.-D.; Stein, T. Engineering *Bacillus subtilis* ATCC 6633 for improved production of the lantibiotic subtilin. *Applied Microbiology and Biotechnology* **2006**, *69*, 532–536, doi:https://www.doi.org/10.1007/s00253-005-0023-9.
44. Brötz, H.; Bierbaum, G.; Markus, A.; Molitor, E.; Sahl, H.G. Mode of action of the lantibiotic mersacidin: Inhibition of peptidoglycan biosynthesis via a novel mechanism? *Antimicrobial Agents and Chemotherapy* **1995**, *39*, 714–719, doi:https://www.doi.org/10.1128/aac.39.3.714.
45. Paik, S.H.; Chakicherla, A.; Hansen, J.N. Identification and Characterization of the Structural and Transporter Genes for, and the Chemical and Biological Properties of, Sublancin 168, a Novel Lantibiotic Produced by *Bacillus subtilis* 168. *Journal of Biological Chemistry* **1998**, *273*, 23134–23142, doi:https://www.doi.org/10.1074/jbc.273.36.23134.
46. Begley, M.; Cotter, P.D.; Hill, C.; Ross, R.P. Identification of a Novel Two-Peptide Lantibiotic, Lichenicidin, following Rational Genome Mining for LanM Proteins. *Applied and Environmental Microbiology* **2009**, *75*, 5451–5460, doi:https://www.doi.org/10.1128/aem.00730-09.
47. Shelburne, C.E.; An, F.Y.; Dholpe, V.; Ramamoorthy, A.; Lopatin, D.E.; Lantz, M.S. The spectrum of antimicrobial activity of the bacteriocin subtilosin A. *Journal of Antimicrobial Chemotherapy* **2007**, *59*, 297–300, doi:https://www.doi.org/10.1093/jac/dkl495.
48. Pattnaik, P.; Kaushik, J.K.; Grover, S.; Batish, V.K. Purification and characterization of a bacteriocin-like compound (Lichenin) produced anaerobically by *Bacillus licheniformis* isolated from water buffalo. *Journal of Applied Microbiology* **2001**, *91*, 636–645, doi:https://www.doi.org/10.1046/j.1365-2672.2001.01429.x.
49. Hammami, I.; Jaouadi, B.; Bacha, A.B.; Rebai, A.; Bejar, S.; Nesme, X.; Rhouma, A. *Bacillus subtilis* bacteriocin Bac 14B with a broad inhibitory spectrum: Purification, amino acid sequence analysis, and physicochemical characterization. *Biotechnology and Bioprocess Engineering* **2012**, *17*, 41–49, doi:https://www.doi.org/10.1007/s12257-010-0401-8.
50. Wong, J.H.; Hao, J.; Cao, Z.; Qiao, M.; Xu, H.; Bai, Y.; Ng, T.B. An antifungal protein from *Bacillus amyloliquefaciens*. *Journal of Applied Microbiology* **2008**, *105*, 1888–1898, doi:https://www.doi.org/10.1111/j.1365-2672.2008.03917.x.

51. Phelan, R.W.; Barret, M.; Cotter, P.D.; O'Connor, P.M.; Chen, R.; Morrissey, J.P.; Dobson, A.D.; O'Gara, F.; Barbosa, T.M. Subtilomycin: A new lantibiotic from *Bacillus subtilis* strain MMA7 isolated from the marine sponge Haliclona simulans. *Marine drugs* **2013**, *11*, 1878–1898.
52. Scholz, R.; Vater, J.; Budiharjo, A.; Wang, Z.; He, Y.; Dietel, K.; Schwecke, T.; Herfort, S.; Lasch, P.; Borriss, R. Amylocyclicin, a novel circular bacteriocin produced by Bacillus amyloliquefaciens FZB42. *J Bacteriol* **2014**, *196*, 1842–1852.
53. Chopra, L.; Singh, G.; Kumar Jena, K.; Sahoo, D.K. Sonorensin: A new bacteriocin with potential of an anti-biofilm agent and a food biopreservative. *Scientific reports* **2015**, *5*, 1–13.
54. Liu, W.-W.; Wei, M.; Zhu, B.-Y.; Du, Y.-C.; Feng, L. Antagonistic activities of volatiles from four strains of Bacillus spp. and Paenibacillus spp. against soil-borne plant pathogens. *Agricultural Sciences in China* **2008**, *7*, 1104–1114.
55. Arguelles Arias, A.; Ongena, M.; Devreese, B.; Terrak, M.; Joris, B.; Fickers, P. Characterization of amylolysin, a novel lantibiotic from Bacillus amyloliquefaciens GA1. *PLoS One* **2013**, *8*, e83037.
56. Chakraborty, K.; Thilakan, B.; Kizhakkekalam, V. Antibacterial aryl-crowned polyketide from Bacillus subtilis associated with seaweed Anthophycus longifolius. *Journal of applied microbiology* **2018**, *124*, 108–125.
57. Romero-Tabarez, M.; Jansen, R.; Sylla, M.; Lünsdorf, H.; Häußler, S.; Santosa, D.A.; Timmis, K.N.; Molinari, G. 7-O-malonyl macrolactin A, a new macrolactin antibiotic from Bacillus subtilis active against methicillin-resistant Staphylococcus aureus, Vancomycin-resistant enterococci, and a small-colony variant of Burkholderia cepacia. *Antimicrobial Agents and Chemotherapy* **2006**, *50*, 1701–1709.
58. Yuan, J.; Raza, W.; Shen, Q.; Huang, Q. Antifungal activity of Bacillus amyloliquefaciens NJN-6 volatile compounds against Fusarium oxysporum f. sp. cubense. *Applied and environmental microbiology* **2012**, *78*, 5942–5944.
59. Arbsuwan, N.; Payoungkiattikun, W.; Sirithorn, P.; Daduang, S.; Jangpromma, N.; Dhiravisit, A.; Hahm, Y.T.; Neubert, L.K.; Klaynongsruang, S. Purification and characterization of macrolactins and amicoumacins from Bacillus licheniformis BFP011: A new source of food antimicrobial substances. *CyTA-Journal of Food* **2018**, *16*, 50–60.
60. MONDOL, M.M.; Ju, L.Y.; Seok, L.J.; Jae, S.H. Macrolactin Antibiotics from a Marine-derived Bacterium *Bacillus* sp. 102CH635-3.**2011**, 281-281.
61. Liu, C.; Lu, J.; Lu, L.; Liu, Y.; Wang, F.; Xiao, M. Isolation, structural characterization and immunological activity of an exopolysaccharide produced by Bacillus licheniformis 8-37-0-1. *Bioresource technology* **2010**, *101*, 5528–5533.
62. Yang, S.; Du, G.; Chen, J.; Kang, Z. Characterization and application of endogenous phase-dependent promoters in Bacillus subtilis. *Applied microbiology and biotechnology* **2017**, *101*, 4151–4161.
63. Chen, H.; Xiao, X.; Wang, J.; Wu, L.; Zheng, Z.; Yu, Z. Antagonistic effects of volatiles generated by Bacillus subtilis on spore germination and hyphal growth of the plant pathogen, Botrytis cinerea. *Biotechnology letters* **2008**, *30*, 919–923.
64. Zimmerman, S.B.; Schwartz, C.D.; Monaghan, R.L.; Pelak, B.A.; Weissberger, B.; Gilfillan, E.C.; Mochales, S.; HERNANDEZ, S.; CURRIE, S.A.; TEJERA, E. Difficidin and Oxydifficidin: Novel broad spectrum antibacterial antibiotics produced by *Bacillus*

subtilis I. Production, taxonomy and antibacterial activity. *The Journal of Antibiotics* **1987**, *40*, 1677–1681.
65. Müller, S.; Strack, S.N.; Hoefler, B.C.; Straight, P.D.; Kearns, D.B.; Kirby, J.R. Bacillaene and sporulation protect *Bacillus subtilis* from predation by Myxococcus xanthus. *Applied and environmental microbiology* **2014**, *80*, 5603–5610.
66. Shimojima, Y.; Hayashi, H.; Ooka, T.; Shibukawa, M.; Iitaka, Y. Studies on AI-77s, microbial products with gastroprotective activity. Structures and the chemical nature of AI-77s. *Tetrahedron* **1984**, *40*, 2519–2527.
67. Okazaki, W.; Akiba, T.; Horikoshi, K.; Akahoshi, R. Production and properties of two types of xylanases from alkalophilic thermophilic Bacillus spp. *Applied microbiology and biotechnology* **1984**, *19*, 335–340.
68. Miwa, H.; Ahmed, I.; Yokota, A.; Fujiwara, T. *Lysinibacillus parviboronicapiens* sp. nov., a low-boron-containing bacterium isolated from soil. *International Journal of Systematic and Evolutionary Microbiology* **2009**, *59*, 1427–1432.
69. Tang, Q.; Yin, K.; Qian, H.; Zhao, Y.; Wang, W.; Chou, S.-H.; Fu, Y.; He, J. Cyclic di-GMP contributes to adaption and virulence of *Bacillus thuringiensis* through a riboswitch-regulated collagen adhesion protein. *Scientific reports* **2016**, *6*, 1–12.
70. Canedo, V.; Benavides, J.; Golmirzaie, A.; Cisneros, F.; Ghislain, M.; Lagnaoui, A. Assessing Bt-transformed potatoes for potato tuber moth, Phthorimaea operculella (Zeller), management. *CIP Program Report* **1997**, *1989*.
71. Calvo, H.; Mendiara, I.; Arias, E.; Gracia, A.P.; Blanco, D.; Venturini, M.E. Antifungal activity of the volatile organic compounds produced by *Bacillus velezensis* strains against postharvest fungal pathogens. *Postharvest Biology and Technology* **2020**, *166*, 111208.
72. Iqbal, S., Vollmers, J., & Janjua, H. A. (2021). Genome mining and comparative genome analysis revealed niche-specific genome expansion in antibacterial bacillus pumilus strain SF-4. *Genes*, *12*(7), 1060.
73. Khan, A.; Gupta, A.; Singh, P.; Mishra, A.K.; Ranjan, R.K.; Srivastava, A. Siderophore-assisted cadmium hyperaccumulation in Bacillus subtilis. *International Microbiology* **2020**, *23*, 277–286.
74. Zhang, D.; Yu, S.; Yang, Y.; Zhang, J.; Zhao, D.; Pan, Y.; Fan, S.; Yang, Z.; Zhu, J. Antifungal effects of volatiles produced by *Bacillus subtilis* against Alternaria solani in potato. *Frontiers in microbiology* **2020**, *11*, 1196.
75. Wang, T.; Liang, Y.; Wu, M.; Chen, Z.; Lin, J.; Yang, L. Natural products from *Bacillus subtilis* with antimicrobial properties. *Chinese Journal of Chemical Engineering* **2015**, *23*, 744–754.
76. Tahir, H.A.S.; Gu, Q.; Wu, H.; Niu, Y.; Huo, R.; Gao, X. *Bacillus* volatiles adversely affect the physiology and ultra-structure of *Ralstonia solanacearum* and induce systemic resistance in tobacco against bacterial wilt. *Scientific Reports* **2017**, *7*, 40481, doi:https://www.doi.org/10.1038/srep40481.
77. Gotor-Vila, A.; Teixidó, N.; Di Francesco, A.; Usall, J.; Ugolini, L.; Torres, R.; Mari, M. Antifungal effect of volatile organic compounds produced by *Bacillus amyloliquefaciens* CPA-8 against fruit pathogen decays of cherry. *Food Microbiology* **2017**, *64*, 219–225, doi:https://www.doi.org/10.1016/j.fm.2017.01.006.
78. Haidar, R.; Roudet, J.; Bonnard, O.; Dufour, M.C.; Corio-Costet, M.F.; Fert, M.; Gautier, T.; Deschamps, A.; Fermaud, M. Screening and modes of action of antagonistic bacteria to control the fungal pathogen *Phaeomoniella chlamydospora* involved in grapevine

trunk diseases. *Microbiological Research* **2016**, *192*, 172–184, doi:https://www.doi.org/10.1016/j.micres.2016.07.003.
79. Xie, S.; Liu, J.; Gu, S.; Chen, X.; Jiang, H.; Ding, T. Antifungal activity of volatile compounds produced by endophytic *Bacillus subtilis* DZSY21 against *Curvularia lunata*. *Annals of microbiology* **2020**, *70*, 1–10.
80. Wu, G.; Liu, S.; Wang, T.; Jiang, Z.; Lv, K.; Wang, Y.; Sun, C. Total synthesis of originally proposed and revised structure of hetiamacin A. *Organic letters* **2018**, *20*, 3566–3569.
81. Giorgio, A.; De Stradis, A.; Lo Cantore, P.; Iacobellis, N.S. Biocide effects of volatile organic compounds produced by potential biocontrol rhizobacteria on *Sclerotinia sclerotiorum*. *Frontiers in microbiology* **2015**, *6*, 1056.
